# Ultrafast laser-scanning time-stretch imaging at visible wavelengths

**DOI:** 10.1038/lsa.2016.196

**Published:** 2017-01-27

**Authors:** Jiang-Lai Wu, Yi-Qing Xu, Jing-Jiang Xu, Xiao-Ming Wei, Antony CS Chan, Anson HL Tang, Andy KS Lau, Bob MF Chung, Ho Cheung Shum, Edmund Y Lam, Kenneth KY Wong, Kevin K Tsia

**Affiliations:** 1 Department of Electrical and Electronic Engineering, The University of Hong Kong, Pokfulam Road, Hong Kong 999077, China; 2 Department of Bioengineering, University of Washington, Seattle, Washington 98195, USA; 3 Department of Mechanical Engineering, The University of Hong Kong, Pokfulam Road, Hong Kong 999077, China

**Keywords:** optical time-stretch imaging, pulse stretching, ultrafast laser scanning

## Abstract

Optical time-stretch imaging enables the continuous capture of non-repetitive events in real time at a line-scan rate of tens of MHz—a distinct advantage for the ultrafast dynamics monitoring and high-throughput screening that are widely needed in biological microscopy. However, its potential is limited by the technical challenge of achieving significant pulse stretching (that is, high temporal dispersion) and low optical loss, which are the critical factors influencing imaging quality, in the visible spectrum demanded in many of these applications. We present a new pulse-stretching technique, termed free-space angular-chirp-enhanced delay (FACED), with three distinguishing features absent in the prevailing dispersive-fiber-based implementations: (1) it generates substantial, reconfigurable temporal dispersion in free space (>1 ns nm^−1^) with low intrinsic loss (<6 dB) at visible wavelengths; (2) its wavelength-invariant pulse-stretching operation introduces a new paradigm in time-stretch imaging, which can now be implemented both with and without spectral encoding; and (3) pulse stretching in FACED inherently provides an ultrafast all-optical laser-beam scanning mechanism at a line-scan rate of tens of MHz. Using FACED, we demonstrate not only ultrafast laser-scanning time-stretch imaging with superior bright-field image quality compared with previous work but also, for the first time, MHz fluorescence and colorized time-stretch microscopy. Our results show that this technique could enable a wider scope of applications in high-speed and high-throughput biological microscopy that were once out of reach.

## Introduction

The escalating need for fast dynamics monitoring and high-throughput screening in a broad spectrum of disciplines, ranging from the biomedical sciences to industrial applications, has fueled continuous advancements in high-speed optical imaging technologies^1, 2, 3, 4, 5, 6, 7^. Among these technologies, optical time-stretch imaging, which was initially developed for high-data-rate all-optical signal processing^8^, is distinct in its ability to enable real-time continuous imaging at an ultrafast frame rate at the MHz scale, orders of magnitude faster than any classical imaging system^5^. Bypassing the use of conventional image sensors or laser-beam scanning for image capture and thus circumventing their intrinsic speed limitations, time-stretch imaging offers the unique potential to fulfill the unmet demand for both speed and throughput in many applications. One notable example is high-throughput single-cell imaging^9, 10, 11, 12, 13^, which is crucial for many areas of biomedical research, for example, cancer biology and regenerative medicine, but remains challenging with existing technologies^14, 15^.

At the heart of time-stretch imaging is its all-optical encoding of image frames in the individual laser pulses in real time, at a frame rate defined by the repetition rate of the ultrafast pulsed laser, which is typically tens of MHz^16, 17^. The encoding process typically involves two interchangeable steps^18^: (1) space-wavelength mapping (also known as spectral encoding), in which the spatial information of the specimen is encoded in the wavelength spectrum of the laser pulse and (2) wavelength-time mapping (also known as dispersive Fourier transformation^19^), in which the pulse is temporally stretched. More precisely, the wavelength spectrum of each pulse is transformed into a temporal waveform through group velocity dispersion (GVD) in a dispersive medium.

In particular, this dual-mapping process, together with its continuous operation in real time, makes this technique appealing for ultrafast all-optical laser-scanning imaging at a scan rate of tens of MHz^20^, surpassing the speed limitations of the existing beam-scanning technologies. For example, traditional galvanometric mirrors, because of their mechanical inertia, can only offer a scan rate of up to 10 kHz^21^, whereas rotating polygonal mirrors can achieve a speed of ~100 kHz^22^. Scanners based on acousto-optic deflectors (AODs) and electro-optic deflectors (EODs), on the other hand, are not subject to the inherent limitations of mechanical inertia and stability and can thus operate at a faster scan rate of up to hundreds of kHz, at the expense of the angular range of beam deflection and the number of resolvable scanned points^23^. However, this speed is still at least two orders of magnitude slower than that of time-stretch-based laser scanning.

Equally important in time-stretch imaging is the spatial resolution, which should not be compromised at its ultrafast imaging rate. To guarantee high spatial resolution, time-stretch imaging requires the use of highly dispersive media^24^ (to achieve a high GVD of >1 ns nm^−1^), which exist predominantly in the optical fiber format, for example, long single-mode fibers^5, 9, 10, 11, 12, 13, 20^, multi-mode fibers^25, 26^ and fiber Bragg gratings^27^. However, high GVD comes at the expense of severe optical loss—there is a fundamental trade-off between dispersion and loss in any classical medium^19^. Hence, time-stretch imaging has remained largely restricted to operation within the low-loss spectral windows of the available fibers, that is, the telecommunication window of ~1550 nm for typical silica fibers^5, 9, 10, 28^. Moderate GVD (<1 ns nm^−1^) in shorter near-infrared regimes, such as 800 nm^13, 20, 29^ and 1000 nm^11, 12, 30^, is also feasible, with the caveat that smaller-core single-mode fibers are vulnerable to pulse distortion due to optical nonlinearity at high optical power. Further translating time-stretch imaging to the visible spectrum or to a shorter-wavelength regime has thus far been impossible, primarily because of the exceedingly high dispersive and scattering losses in fibers at these wavelengths. However, this short-wavelength window has long been the most critical regime for various techniques of optical microscopy that are widely used in basic biology and biomedical diagnostics. As a result, the potential of high-quality time-stretch imaging for these applications remains elusive.

To address this long-standing challenge, we here present a new strategy for pulse stretching, termed free-space angular-chirp-enhanced delay (FACED), that offers a unique solution for time-stretch imaging in the short-wavelength regime. Not only can it generate enormous and low-loss pulse stretching, the device geometry of FACED also naturally enables an all-optical beam-steering mechanism for ultrafast laser-scanning imaging. In contrast to conventional time-stretch imaging, FACED-based time-stretch imaging offers three distinctive features: (1) Its free-space pulse-stretching operation mitigates the fundamental optical-loss limitation associated with material dispersion and enables a significantly larger dispersion-to-loss ratio than can be achieved with dispersive fibers, especially in the visible spectrum, where silica fibers suffer from severe dispersive and scattering losses. (2) The pulse-stretching concept used in FACED is based on wavelength-invariant geometrical optics and thus does not necessarily entail chromatic dispersion. This introduces a new paradigm of time-stretch imaging, which can now be implemented both with and without spectral encoding. (3) Pulse stretching in FACED simultaneously provides an ultrafast all-optical laser-beam scanning mechanism. Together with its actively reconfigurable pulse-stretching operation, FACED could enable diverse high-speed imaging applications that have long been inaccessible with time-stretch imaging. To illustrate this unique potential, we here demonstrate FACED-enabled ultrafast high-quality time-stretch imaging at ~700 nm. Specifically, we demonstrate MHz fluorescence and colorized time-stretch microscopy for the first time in this unexploited visible-light regime.

## Materials and methods

### Working principles of FACED

**Figure 1:**
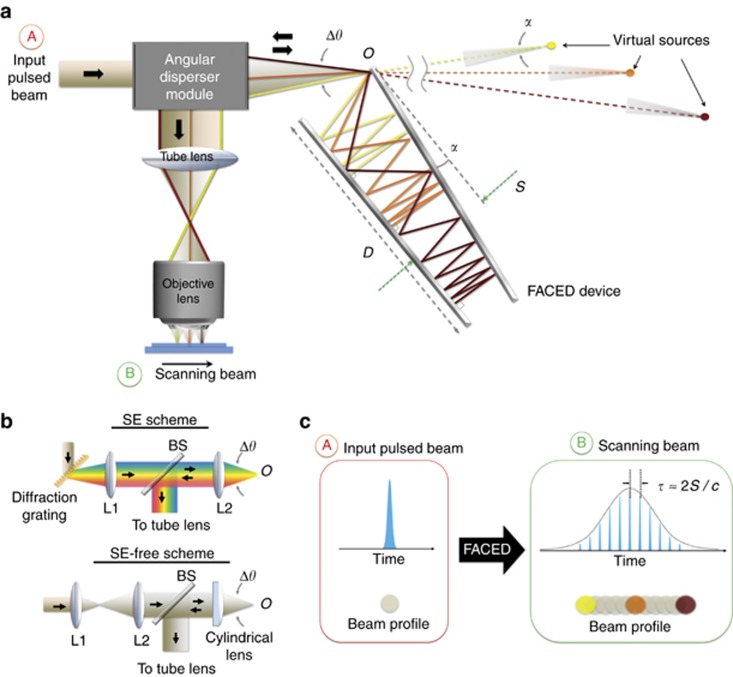
Working principle of FACED. (**a**) Overall schematic illustration of the laser-scanning time-stretch imaging system based on FACED. The input pulsed beam, passing through the angular disperser module (see the detailed schematics in **b**), is focused at the entrance *O* of the FACED device, which consists of a pair of high-reflectivity, angle-misaligned plane mirrors. Because of the minute angle *α*, the beam propagation can be treated as a set of spatially chirped zig-zag paths, which eventually return to the input instead of escaping from the far end of the device. Such a light trajectory substantially enhances the time delays between different paths and thus generates enormous pulse stretching (or temporal dispersion, if the SE scheme is employed). For input rays with incidence angles equal to integer multiples of *α* at the entrance *O*, they return precisely along their initial incoming paths after reaching one of the mirrors at normal incidence (see the three highlighted rays). These rays, called cardinal rays, can be regarded as the light emerging from virtual sources, each of which has a small diverging angle (=*α*). In effect, FACED generates an array of time-delayed virtual sources that are aligned in space and are then projected by the relay-lens system (for example, a tube lens and an objective lens) onto the specimen as a scanning beam. (**b**) Schematic diagrams of the angular dispersers employed in (top) the SE scheme and (bottom) the SE*-*free scheme. SE, with spectral encoding; SE-free, without spectral encoding. The key elements in the SE and SE*-*free schemes that introduce the ‘angular dispersion’ are the diffraction grating and the cylindrical lens, respectively. The telescopic lens systems (consisting of lenses L1 and L2) in both schemes are used to control the input light cone angle Δ*θ*. BS, beam-splitter. (**c**) Overall concept of FACED. FACED not only can introduce pulse stretching in time (with a stretched pulse envelope consisting of a train of sub-pulses) but also transforms an input pulsed beam into an all-optical scanned beam in space. Note that each sub-pulse corresponds to a virtual source, that is, a scanned spot on the specimen (see the three highlighted spots, which correspond to the three cardinal rays in **a**).

Although free space is typically regarded as an ineffective medium for generating sizable dispersion (or pulse stretching), FACED harnesses the minute mirror misalignment angle *α* (typically ~10^−3^ rad) between a pair of highly reflective plane mirrors (separated by a distance *S*), which results in a substantial enhancement in the light-path length and, consequently, the pulse stretching in free space within a practically compact setup (Figure 1a). The input light is required to be a converging pulsed beam focused at the entrance to the FACED device, *O.* This is achieved using an optical module consisting of a relay-lens system and an ‘angular disperser,’ which can be either a diffraction grating (for time-stretch imaging with spectral encoding, called the SE scheme) or simply a focusing cylindrical lens (for time-stretch imaging without spectral encoding, called the SE-free scheme; Figure 1b).

On the basis of ray tracing, the beam propagation in the FACED device can be viewed as a set of spatially chirped zig-zag paths (see the three highlighted paths in Figure 1a) that cannot escape from the far end of the mirror pair but instead must eventually return to the device entrance *O*. This condition is made possible with a proper misalignment-angle geometry and suitable mirror dimensions (see the detailed ray-tracing model in the Supplementary Figs. S1–S5). More importantly, this misaligned mirror geometry creates the spatially chirped paths and substantially enhances the temporal delay of each path within the device in free space. In essence, the output pulse is temporally stretched by this angle-dependent delay. When the input light is a broadband pulsed beam and is angularly dispersed by the diffraction grating in the SE scheme, the zig-zag paths are spectrally encoded and thus introduce significant wavelength-dependent temporal delays (that is, GVD), even in free space.

Specifically, a discrete set of light paths return exactly along their initial incoming paths after they have reached one of the mirrors at normal incidence. These rays, referred to as cardinal rays, have angles of incidence of *θ=kα*, where *k* is an integer, at the entrance *O* (Supplementary Fig. S1). The rays that are not cardinal rays are also back-reflected, although not along their original paths. In effect, these ‘non-cardinal’ rays, along with the cardinal rays, can be regarded as having been emitted from a set of virtual sources, each with a beam divergence angle of *α* (Figure 1a, Supplementary Fig. S5). The FACED device can thus alternatively be regarded as an array of time-delayed virtual sources that are automatically aligned in space by the misaligned mirror geometry. One can then employ any relay-lens system to sequentially project these virtual sources onto various locations on the specimen plane, that is, FACED can provide not only pulse stretching in free space but also, simultaneously, an all-optical laser-scanning mechanism (Figure 1b)—a feature that distinguishes FACED from all previously adopted pulse stretchers for time-stretch imaging.

On the basis of our ray-tracing model, which is applicable to both the SE and SE-free schemes, the number of virtual sources (that is, cardinal rays) *M* is simply proportional to the input cone angle of the beam Δ*θ*:


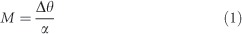


The temporal delay between any two virtual sources can be directly derived from the difference in their optical paths:


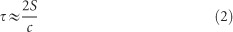


where *c* is the speed of light. Therefore, the input light is ‘redistributed’ in time into a train of sub-pulses, each of which corresponds to a virtual source (Figure 1c). The envelope of the pulse train is the output stretched pulse. The total time delay across all *M* virtual sources, that is, the overall stretched pulse width Δ*T*_total_, can be found to be


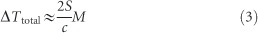


The approximations in Equations (2) and (3) are valid when Δ*θ* is small (<100 mrad, relative error <0.2%). For the SE scheme, we can further quantify the GVD in terms of wavelength, that is, the dispersion parameter (typically in ns nm^−1^), which is defined as *D*_*λ*_*=∂τ/∂λ*. Therefore, for a given source bandwidth Δ*λ* (in wavelength), the total GVD can be evaluated as (Supplementary Fig. S3)


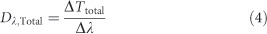


Based on Equations (1)–(4), the geometrical parameters of the device provide multiple degrees of freedom with which to manipulate the total time delay (or GVD), the number of virtual sources, and the time delay between adjacent sources (Supplementary Figs. S2 and S4). In other words, the virtual sources are reconfigurable in both their spatial and temporal positions. For the typical dimensions of a FACED device, for example, *S*=25 mm, a tunable *α*=0.5–1 mrad, and an input light cone angle of Δ*θ*<50 mrad, one can achieve a reconfigurable total delay Δ*T*_total_ ranging between 10 and 15 ns for *M*~60–90. For a typical bandwidth for time-stretch imaging of 10 nm^24^, we can equivalently achieve a large and reconfigurable GVD of *D*_*λ*_,_total_~1–1.5 ns nm^−1^ in the SE scheme, regardless of the center wavelength of the source.

In FACED, the mirror reflectivity is the main factor determining the intrinsic loss and therefore must be high to guarantee low loss and a large time delay (or a large GVD). This is readily achievable with commercially available dielectric multilayer mirrors, which can provide high reflectivity (>99.5%) with a customized bandwidth (~100 nm or wider) centered on a range of wavelengths from visible to infrared (~0.4–2 μm). Therefore, FACED devices are superior to fiber-based devices for operation with large-scale and tunable time delay or dispersion outside the telecommunication band, where fiber losses are impractically high.

Importantly, FACED devices should not be viewed as similar to the classical chirped mirrors configuration^31^, a virtually imaged phased array^32^, or a Gires–Tournois interferometer^33^ because its operation is entirely based on the geometrical ray tracing of the spatially chirped paths and involves neither phase matching nor the Bragg reflection concept in any way.

To perform time-stretch imaging using FACED, the virtual sources are first imaged near the common focal plane by the coupling lens (L2 in the SE scheme or the cylindrical lens in the SE*-*free scheme) (Figure 1b) and are then relayed to the focal plane of the microscope, generating the one-dimensional (1D) all-optical scanning beam (Supplementary Figs. S6–S9). The distance between adjacent spots (*V*_*d*_) imaged at the focal plane can be calculated as follows:


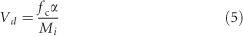


where *f*_*c*_ is the focal length of the coupling lens and *M*_*i*_ is the magnification of the microscope. Through tuning of the misalignment angle *α*, *V*_*d*_ can be tuned to be less than half of the Airy spot size of the microscope to achieve diffraction-limited resolution. Given an input light cone angle Δ*θ*, the field of view (FOV) of the scanner is given by


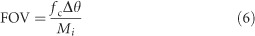


The input cone angle Δ*θ* can be manipulated by the two-lens telescopic system to match the numerical aperture (NA) of the FACED device (Supplementary Fig. S1) such that all of the laser power can be used. We can also introduce a slit at the common focal plane to flexibly control the input cone angle Δ*θ*, at the cost of some laser power. In this work, the latter approach was used to control Δ*θ* and, hence, the field of view.

### Experimental setup

A FACED device was constructed using a pair of mirrors with high reflectivity mainly in the visible spectrum (~700 nm). We also conducted performance tests in the near-infrared range (1064 and 1550 nm) to illustrate the wavelength-insensitive operation of the device (Supplementary Fig. S10). Specifically, three customized dielectric mirror pairs (ios Optics), all with high reflectivity (>99.5%) in broadband (>100 nm) reflection spectra centered at 1550, 1064 and 710 nm, respectively, were used. The rectangular mirrors, with dimensions of 25 × 200 mm, were mounted on kinematic mounts (Thorlabs Inc., Newton, NJ, USA). The separation of the mirror pair was controlled using a linear translational stage (Thorlabs Inc.), and the mirror misalignment angle was adjusted using a high-precision rotational stage (Newport Corporation, Irvine, CA, USA) to provide tuning of the pulse-stretching characteristics. For the SE scheme, a diffraction grating (1800 lines per mm) was employed as the angular disperser. For the SE*-*free scheme, the angular disperser was simply a cylindrical lens. In both the SE and SE*-*free schemes, a telescopic 4-*f* relay-lens system was used to manipulate the converging angle input to the FACED device (Figure 1b). The total illumination intensity on the imaging plane in the SE scheme (SE*-*free scheme) was ~9 kW cm^−2^ (~17 kW cm^−2^), whereas the energy fluence of a single pulse was ~0.1 mJ cm^−2^ (~0.2 mJ cm^−2^). Along the return path, the stretched pulsed beam on the Fourier plane of the FACED device’s entrance point *O* was relayed by a tube lens and an objective lens onto the specimen plane for laser-scanning time-stretch imaging. Equivalently, these lenses projected the virtual sources onto the specimen plane. A beam splitter (50:50) was used to separate the output beam from the input beam. The signals in all experiments were collected using a high-speed photodetector (bandwidth >9 GHz) and a real-time oscilloscope (bandwidth: 4–20 GHz; sampling rate: 20–80 GSa s^−1^). The detailed configurations for the experiments are described in the Supplementary Figs. S11 and S12; Supplementary Table S1.

### Methods of image reconstruction

Each captured time-stretch waveform, essentially a 1D line scan, is normalized with respect to the background waveform captured with no specimen present. The final two-dimensional (2D) image is formed by digitally aligning the consecutive waveforms along the slow axis (that is, the microfluidic flow direction or the sample stage scanning direction). Notably, the sub-pulse train within the overall time-stretch waveform is smoothed by a digital low-pass filter (for example, a boxcar window filter) or simply a slower digitizer to extract the key features of the envelope of the waveform without sacrificing spatial resolution.

Regarding the digital colorization of the time-stretch image, a colorization algorithm based on a pre-trained library was used, in which each grayscale value was conditionally mapped to specific red–green–blue values for pixel-to-pixel multi-color visualization^34^. Multiple images of the same features on similar tissue captured using both the time-stretch microscope and a commercial light microscope (Eclipse Ni-U, Nikon Corporation, Minato, Tokyo, Japan) were pre-fed into a custom MATLAB routine for the establishment of the mapping library, which was optimized for the dyes used in the experiment, that is, fast red and methylene blue. The mapping library was also further processed via first-degree extrapolation and interpolation to reduce colorizing error due to missing values in the library. During colorization, a mask was also calculated based on thresholding the monochromatic FACED image to isolate voids, which correspond to cell bodies with lower optical attenuation. Because the cell nucleus is located within the cell body, it is safe to assume that fast red can only stain features inside cell bodies. Therefore, separate libraries were pre-trained for the extracellular matrix and the cell bodies, which were predominantly stained by either methylene blue dye or fast red dye, respectively. These libraries were applied to the FACED images separately as dictated by the mask for optimal visualization.

### Microfluidic channel fabrication

The polydimethylsiloxane (PDMS)-based microfluidic channel chip used in the flow experiments was fabricated using standard replica molding methods. The elastomer base was first mixed with the curing agent at a ratio of 10:1 (w/w) (Sylgard 184, Dow Corning Corp., Midland, MI, USA), and the mixture was then poured onto a premade photoresist-silicon wafer stamp. Next, the liquid prepolymer replica was cross-linked by means of baking at 65 °C and peeled off. The cured plastic was lifted off of the wafer. Inlet and outlet holes were created using a biopsy punch, followed by further incubation of the semi-product in an incubator (Thermo Fisher Scientific, Waltham, MA, USA) at 65 °C for 3 h. Both the molded PDMS slab and a glass slide were cleaned and treated with oxygen plasma using a plasma cleaner (Harrick Plasma Inc., Ithaca, NY, USA) for 2 min. Afterward, the treated components were covalently bonded, and the vacant space in the replicated pattern formed the microfluidic channel. Polytetrafluoroethylene tubing (Scientific Commodities Inc., Lake Havasu City, AZ, USA) was inserted into the punched holes, and additional PDMS prepolymer was added around the edges of the PDMS slab to seal any gaps. The newly fabricated chips were heated at 80 °C overnight before the experiment. The microfluidic channel geometry and dimensions were designed to allow ultrafast microfluidic flow under the inertial flow focusing mechanism^35^.

### Cell culture and preparation

THP-1 cells (human monocytic leukemia cell line) were cultured in Roswell Park Memorial Institute (RPMI) medium 1640 (Gibco, Thermo Fisher Scientific) supplemented with 10% fetal bovine serum (Hyclone, Thermo Fisher Scientific), 1% penicillin streptomycin (Gibco, Thermo Fisher Scientific) and 1% 2 mM Glutamax (Gibco, Thermo Fisher Scientific) at 37 °C, 95% humidity and 5% CO_2_ until confluence was achieved. Optimal conditions were maintained before the experiment. Fresh blood samples, in volumes of ~3 ml, were drawn from the left median cubital artery of a healthy donor and were then maintained at 2–8 °C in an ethylene diamine tetraacetic acid (EDTA) anticoagulant evacuated tube (Greiner Bio-One GmbH, Kremsmünster, Austria). Peripheral blood mononuclear cells (PBMCs) and red blood cell (RBCs) were extracted from the blood samples using a standard density-gradient centrifugation technique for the experiment. Blood was collected at least 8 h prior to the experiment. The living microphytoplankton *Scenedesmus acutus* (Carolina Biological Supply Company, Burlington, NC, USA) was cultured in a freshwater medium under room light before the experiment.

## Results and discussion

### Basic performance of FACED

Pulse stretching in FACED is achieved entirely by virtue of the misaligned mirror geometry, which creates large-scale and reconfigurable time delays between the cardinal rays (virtual sources). The working principle is fundamentally based on wavelength-invariant ray tracing and can thus be implemented regardless of whether the SE or SE-free scheme is chosen. For the sake of argument, we experimentally verified the pulse-stretching performance (for example, the scale, reconfigurability and pulse-stretching loss) based primarily on the SE scheme at visible wavelengths (~700 nm). This demonstration is pertinent for illustrating the unique capability of FACED that is absent in dispersive fibers—that is, enormous GVD with low loss outside the telecommunication band.

**Figure 2:**
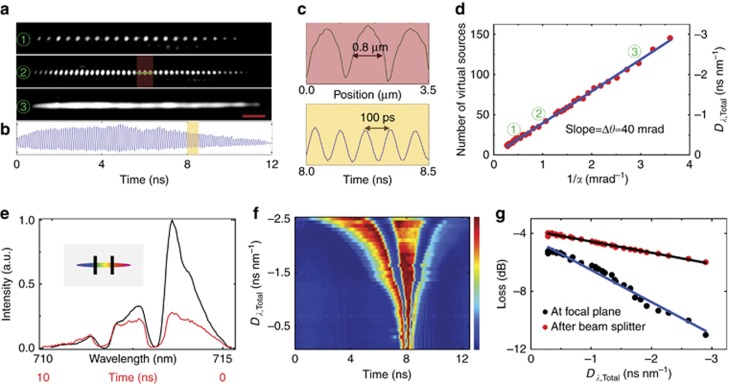
Basic performance of FACED based on the SE scheme. (**a**) Images of three different patterns of scanned spots generated by FACED, that is, the projection of the virtual source arrays onto the focal plane of the objective lens at three mirror misalignment angles *α*. In general, the number of scanned spots and the spot density increase from case 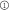 to case 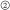 to case 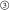. Scanning-beam pattern 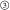, which is a continuous line-beam profile, was used for the time-stretch imaging performed in this work. The scale bar represents 5 μm. (**b**) Time-stretched waveform, which consists of a train of sub-pulses, corresponding to scanning-beam pattern 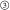. (**c**) (Top) Zoomed-in view of the intensity profile along the green line (red area) in 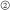. (Bottom) Zoomed-in view of the temporal profile in the yellow area of **b**. (**d**) Reconfigurability of the number of virtual sources, and thus the dispersion (*D*_*λ,*Total_), by varying the mirror misalignment angle *α*. The red dots represent the measured data, and the blue line shows the linear fit to the data. The slope of the fit corresponds to the input light cone angle Δ*θ*. Cases 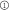, 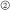 and 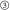 depicted in **a** are highlighted in the plot. (**e**) Single-shot time-stretched waveform (red) and the corresponding spectrum (black) measured using a conventional spectrometer with a total dispersion of –2 ns nm^−1^. Notably, the spectrum was shaped by placing two fiber needles at the intermediate conjugate plane of the virtual sources (to generate two intensity dips in the spectrum). (**f**) Evolution of the temporal profile of the stretched pulse within the range of normal dispersion (that is, *D*_*λ,*Total_=–200 ps nm^−1^ to –2.5 ns nm^−1^). The dispersion can be tuned in both the normal and anomalous dispersion regimes (Supplementary Fig. S14). (**g**) Dependence of the losses measured at the focal plane and after the beam splitter (BS, refer to Figure 1b) on the total dispersion.

Among all geometrical parameters of the FACED device, the mirror misalignment angle *α* is a particularly effective means of manipulating the pulse-stretching performance (Supplementary Figs. S2 and S4). First, the number of virtual sources *M*, that is, the number of resolvable scanned spots, can be flexibly varied within a small range of *α*~1 mrad (Figure 2a). Again, the time-stretched waveform consists of a train of sub-pulses, each of which corresponds to a virtual source (Figure 2b). The time between neighboring sub-pulses is measured to be *τ*~100 ps (Figure 2c), which is consistent with Equation (2). By decreasing *α*, we can increase the number of scanned spots within the field of view such that a continuous intensity profile of the line-scan beam is achieved (Figure 2a)—a favorable condition for typical laser-scanning imaging applications. Note that the size of the scanned spots is ~0.8 μm (Figure 2c), close to the diffraction limit, as shown in our numerical analysis (Supplementary Fig. S9). Similar performance can be achieved under the SE*-*free scheme (Supplementary Figs. S7 and S13).

More importantly, we demonstrated that an enormous reconfigurable GVD can be generated between –200 ps nm^−1^ and –2.5 ns nm^−1^ at visible wavelengths (~700 nm, bandwidth ~5 nm) by changing the mirror misalignment angle (Figure 2d). This behavior is accurately predicted by our ray-tracing model (that is, Equations (3) and (4)). Single-shot wavelength-to-time mapping at an ultrafast rate of 80 MHz was also confirmed by a measurement using a classical spectrometer (Figure 2e). The spectral shape is generally preserved while the GVD is varied over a large range (Figure 2f). This large-scale GVD tunability can even be switched between normal and anomalous GVD (Supplementary Fig. S14).

The key significance of FACED is its free-space operation, which enables enormous pulse stretching or GVD at low loss by bypassing the material loss associated with high dispersion in a fiber-based pulse stretcher. In FACED, wider pulse stretching (that is, higher GVD) leads to higher loss. This can be easily understood from the fact that the pulse stretching scales with the number of mirror reflections and, thus, the total reflectivity loss. Nevertheless, the intrinsic loss remains reasonably low (~4–6 dB) even for quite large GVD (0.3–3 ns nm^−1^) (Figure 2g). An additional 2–6 dB transmission loss is introduced by the objective lens. The unique advantage of FACED becomes even clearer when we compare the loss as a function of GVD for the FACED device with that for silica fibers. Specifically, for every 1 ns nm^−1^ increase in GVD, FACED introduces an additional loss of only ~1 dB at 700 nm. This is in stark contrast to the fiber case, in which a prohibitively high loss of~50 dB is introduced. Although optical amplification is generally essential to compensate for the dispersive loss in time-stretch operation^16, 18, 19^, the low-loss characteristics of FACED intriguingly make optical amplification less critical, even at high GVD. Because optical amplifiers that operate in the visible wavelength regime and are compatible with time-stretch imaging are not readily available and usually necessitate a bulky system architecture (for example, an optical parametric amplifier^36^), this relaxation of the requirement for optical amplification thus renders FACED a unique approach for efficient time-stretch imaging in this once-forbidden short-wavelength window.

Robust control of the pulse stretching and GVD over a sub-degree tuning range of mirror misalignment angles is made practical by the wide availability of optomechanical mirror mounts, which provide superior kinematic stability. With our configuration fabricated using off-the-shelf optomechanical parts, a mechanical angular resolution of ~15 μrad could be achieved in our experiments. We also confirmed that the entire FACED device showed a high degree of robustness in terms of long-term power (2% (s.d./mean)) and GVD stability (2.4% (s.d./mean)) during continuous operation over 60 min (Supplementary Fig. S15).

### FACED-based time-stretch imaging (SE scheme)

**Figure 3:**
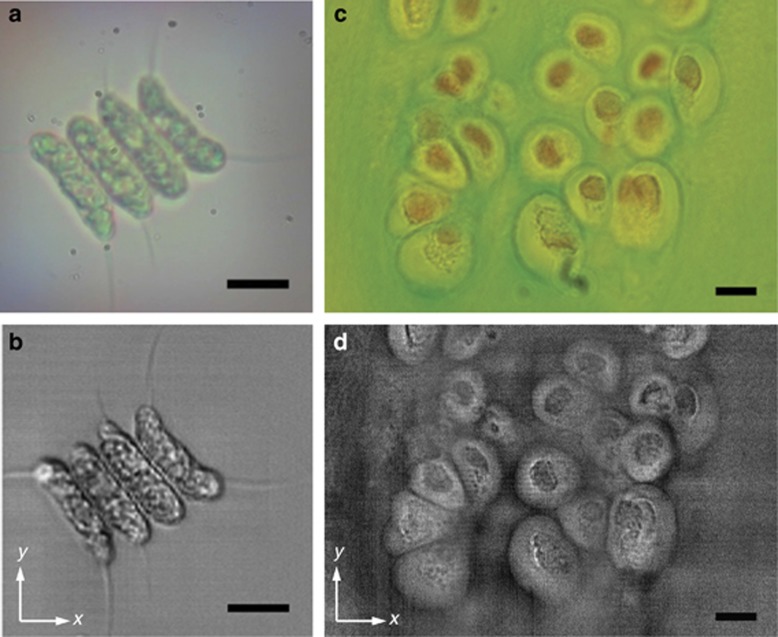
FACED-based bright-field time-stretch microscopy at 710 nm of living cells and tissue sections. (**a**, **b**), Live *Scenedesmus acutus* cells as captured by **a** a home-built bright-field transmission microscope (Supplementary Fig. S11) and **b** the FACED-based time-stretch microscope. (**c**, **d**) Knee tissue section stained with alcian blue captured by **c** an ordinary bright-field transmission microscope (Eclipse Ni-U, Nikon) and **d** the FACED-based time-stretch microscope. The time-stretch images were captured by line scanning the specimen along the *y* axis (slow axis) with a line-scan rate of 10 MHz along the *x* axis (fast axis). The imaging objectives used had numerical apertures of NA=0.66 for **a**, **b**, **d** and NA=0.75 for **c** Scale bars=10 μm.

We first evaluated the basic imaging performance based on the visible-light bright-field time-stretch imaging of stained biological tissue sections (knee tissue sections) and live microphytoplankton (*Scenedesmus acutus*) at an effective line-scan rate of 10 MHz (Figure 3). The time-stretch image quality is generally comparable to that achieved by an ordinary bright-field transmission microscope, but it is achieved at an ultrafast MHz scanning speed. In our current imaging system operating at ~700 nm, with the mirror misalignment angle set to smaller than ~1 mrad, we have 120 virtual sources packed within a field of view of 50 μm (Figure 2a and 2b). On the basis of the Nyquist sampling criterion, the spatial resolution is estimated to be ~0.8 μm, which is larger than the diffraction limit, that is, ~0.65 μm, for an objective lens with NA=0.66. By contrast, the diffraction-limited resolution for time-stretch microscopy at 1550 nm is 1.4 μm with the same objective lens. Moreover, the resolution could be pushed to the diffraction limit if a smaller field of view were targeted. We also compared the time-stretch imaging performance between FACED and a dispersive-fiber-based configuration (Supplementary Fig. S16). Note that this comparison was performed at 1060 nm instead of 700 nm because of the exceedingly high fiber loss at visible wavelengths. Both systems, that is, the FACED-based and fiber-based time-stretch imaging systems, could resolve the smallest element of group 8 of resolution target USAF-1951, which had a line width of 1.1 μm. Clearly, FACED-based time-stretch imaging is an effective modality comparable to fiber-based time-stretch imaging.

**Figure 4:**
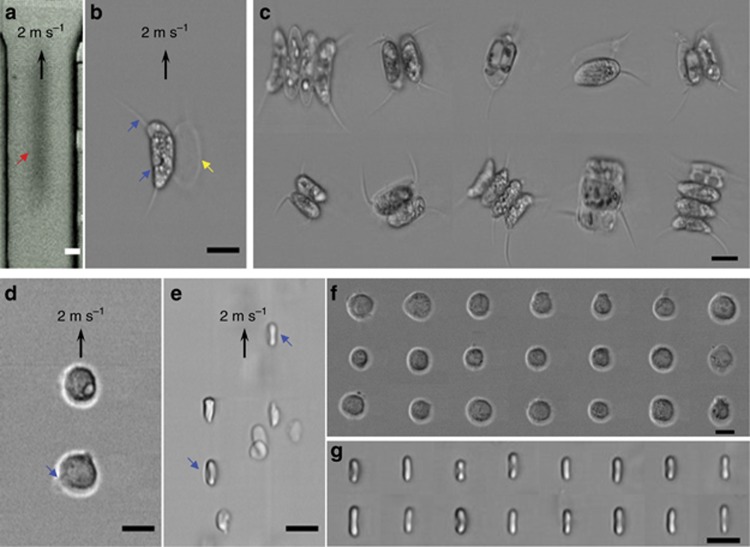
FACED-based time-stretch microscopy in an ultrafast microfluidic flow at 710 nm. (**a**, **b**) Images of *Scenedesmus acutus* in an ultrafast microfluidic flow captured by **a** a CMOS camera (15 000 f.p.s.) and **b** the FACED-based time-stretch microscope. The blur-free images reveal the subcellular structure, for example, vacuoles and long filaments (blue arrows), and the cell membrane of the non-pigmented *Scenedesmus acutus,* which is essentially detritus (yellow arrow). (**c**) Imagery of *Scenedesmus acutus* captured by the time-stretch microscope. (**d, e**) Time-stretch images of THP-1 and human red blood cells (RBCs) in an ultrafast microfluidic flow. Fine features, for example, blebs and the biconcave shapes of the RBCs, are visualized (blue arrows). (**f**, **g**) Imagery of THP-1 and RBCs captured by the time-stretch microscope. The imaging objectives used had numerical apertures of NA=0.25 for **a** and NA=0.66 for all time-stretch images. The scale bars represent 10 μm.

By leveraging the 1D line-scan imaging operation, we also demonstrated single-cell visible-light time-stretch imaging in an ultrafast microfluidic flow (~2 m s^−1^) at a line-scan rate of 80 MHz (Figure 4). We again note that the flow direction (slow axis) is orthogonal to the line-scan direction (fast axis). Under the assumption of a typical cell size of ~10 μm and an average separation of 20 μm, this is equivalent to an imaging throughput of as high as 100 000 cells per second—one to two orders of magnitude faster than state-of-the-art imaging flow cytometers^37^. Compared with the image captured by a high-speed camera (15 000 f.p.s.; Figure 4a), the time-stretch images of *Scenedesmus acutus* cells (Figure 4b and 4c), monocytic leukemia cells (THP-1; Figure 4d), and human red blood cells (Figure 4e) not only are blur-free but also exhibit high image contrast and resolution, revealing sub-cellular features. For example, the FACED-based time-stretch imaging of the microphytoplankton reveals the vacuoles and spines as well as the shell of a dead cell (Figure 4b). Fine features, for example, the blebs of the THP-1 and the biconcave shapes of the RBCs, are also clearly visualized (Figure 4d and 4e). Additional images of THP-1 and RBCs captured by the time-stretch microscope are also shown (Figure 4f and 4g). It should be emphasized that such high image quality has not been achieved with any previous time-stretch imaging modality. In combination with the increased compatibility of FACED-based time-stretch imaging with fluorescence imaging due to its ability to operate in the visible light regime (as discussed later), the image quality achievable with the proposed technique endows it with immense potential for high-throughput imaging flow cytometry.

**Figure 5:**
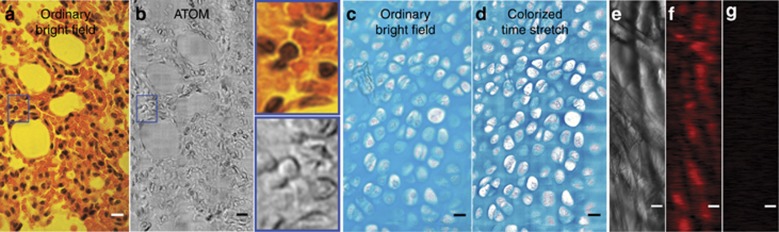
Various FACED-based visible-light time-stretch imaging modalities (asymmetric-detection time-stretch microscopy (ATOM), colorized and fluorescence imaging). (**a**, **b**) Ordinary bright-field transmission and ATOM images of a hematoxylin-and-eosin (H&E)-stained lung tissue section. The two insets show the corresponding zoomed-in views of the areas indicated by blue rectangles in **a** and **b**. (**c**, **d**) Ordinary bright-field transmission and colorized time-stretch microscopy images of a stained cartilage tissue section (stained with methylene blue and fast red). (**e, f**) Ordinary bright-field transmission and fluorescence time-stretch microscopy images of tissue paper stained with a fluorescent dye (Antibody-CF750 conjugates, 100 μg ml^−1^). (**g**) Fluorescence time-stretch microscopy image of stain-free tissue paper. The imaging objectives used had numerical apertures of NA=0.75 for **a** and **c** and of NA=0.66 for **e** and all time-stretch images. Scale bars=10 μm.

We also demonstrated other visible-light time-stretch imaging modalities enabled by FACED. First, by tilting the illuminating beam, the system can readily be made to perform asymmetric-detection time-stretch optical microscopy at 700 nm^12^, which yields a pseudo-three-dimensional image with enhanced phase-gradient image contrast (Figure 5a and 5b)—an important attribute for high-contrast label-free imaging applications. Second, by accessing the visible spectrum, color time-stretch imaging is made possible for the first time through digital colorization, which transforms the grayscale time-stretch image amplitudes (that is absorption contrasts) into unique colors through a learning algorithm^34^. A colorized time-stretch image of a stained cartilage tissue section (stained with methylene blue and fast red) shows image quality consistent with that of ordinary bright-field transmission microscopy (Figure 5c and 5d) but acquired at an ultrafast line-scan rate of 10 MHz. By contrast, colorization is ineffective for optical time-stretch imaging performed in the near-infrared because the absorption spectra of differently colored dyes typically show insignificant differences outside the visible-light band. This colorization capability has immediate application in ultrahigh-throughput whole-slide imaging for digital histopathology^38^ when combined with a high-speed specimen scanning stage.

Optical time-stretch imaging with FACED at visible wavelengths also overcomes the long-standing barrier preventing the realization of fluorescence time-stretch imaging—an integral tool for basic biology and biomedical diagnostics. To confirm this claim, we employed the same setup used for bright-field time-stretch imaging with the exception of an additional filter to ensure the collection of only the fluorescence emissions (Supplementary Fig. S11). We thus demonstrated the first fluorescence time-stretch imaging (excitation at 710 nm, emission at ~770 nm) at an ultrafast line-scan rate as high as 1.25 MHz (Figure 5e and 5f). The fluorescence signal was verified through comparison with an image of a specimen without the fluorescence dye (Figure 5g). Note that the ultrafast scanning speed achieved here represents a new regime of laser-scanning fluorescence imaging in which the dwell time of the scanned spot (~100 ps) is comparable to (or shorter than) both the typical fluorescence lifetime (>1 ns) and the detection response time of the system (~2 ns instrument response time of the photomultiplier tube in the system), both of which must be considered when evaluating the spatial resolution, according to our previous study^39^. In other words, there is cross-talk between adjacent scanned points in this ultrafast scanning regime. Such cross-talk can be minimized through the use of a higher-speed, sensitive photodetector, for examples, a hybrid photodetector (with a response time of hundreds of ps). With a lower-repetition-rate laser (<10 MHz), this effect can also be mitigated by adjusting the mirror separation in the FACED device such that the time delay between two adjacent scanned spots is comparable to the fluorescence lifetime (that is, a wider mirror separation; see Supplementary Fig. S2).

We note that FACED-based time-stretch imaging using the SE scheme is essentially an implementation of chromo-modal dispersion (CMD)^25^. In CMD, pulse stretching is induced by the combined effect of chromatic dispersion and the modal dispersion of different spatial modes confined in a multimode waveguide. The time delay between cardinal rays in FACED is analogous to the modal dispersion in CMD. However, the pulse stretching in FACED is entirely predicted by the path-length-induced time delay, i.e., chromatic dispersion is not a necessary condition. Moreover, instead of requiring high-refractive-index multimode waveguides, FACED relies on its free-space operation and thus has a three-fold advantage: (1) it experiences no dispersive or scattering losses such as those observed in the material of a waveguide/fiber; (2) it is not subject to modal coupling, which was found to limit the tunability of dispersion and impose an upper bound on the achievable pulse stretching in a previous demonstration of CMD^25^; and (3) the spatial profile of the input beam is generally preserved after the application of FACED, allowing the generation of an array of spatially aligned, time-delayed virtual sources. This last feature makes FACED advantageous for laser-scanning imaging applications that cannot be achieved using fiber-based CMD because the beam profile at the fiber output is drastically altered by the spatial overlap of the large number of fiber modes, resulting in a highly speckled output beam.

### FACED-based time-stretch imaging (SE-free scheme)

**Figure 6:**
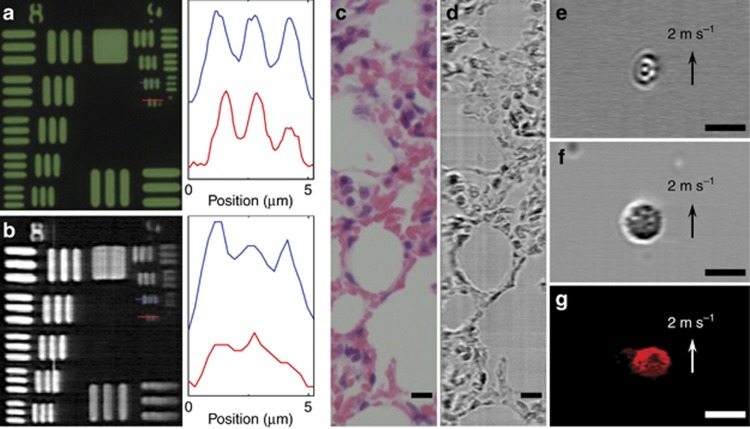
FACED-based time-stretch microscopy at 710 nm using the SE-free scheme. (**a, b**) Ordinary bright-field transmission and time-stretch microscopy images of resolution target USAF-1951. The right insets show the corresponding intensity profiles along the blue and red lines. (**c**, **d**) Ordinary bright-field transmission and time-stretch microscopy images of a hematoxylin-and-eosin (H&E)-stained lung tissue section. (**e**, **f**) Time-stretch images of RBCs and PBMCs in a microfluidic flow. (**g**) Fluorescence time-stretch microscopy image of a 10 μm fluorescent bead in a microfluidic flow. The imaging objectives used had numerical apertures of NA=0.4 for **c** and NA=0.75 for **a** and all time-stretch images. The bright-field time-stretch imaging line-scan rate was 80 MHz, and the fluorescence time-stretch imaging line-scan rate was 8 MHz. Scale bars=10 μm.

We also briefly demonstrated laser-scanning time-stretch imaging based on the SE-free scheme at ~700 nm (Figure 6 and Supplementary Fig. S12). Our experiments show that this time-stretch imaging modality also yields an image resolution comparable to that achieved with an ordinary bright-field transmission microscope. The slightly lower resolution of the FACED images (see the smallest element of group 9 of resolution target USAF-1951 as shown in Figure 6a and 6b) is attributed to the smaller number of scanned spots (that is, *M*~70). This can be further optimized by adjusting both the mirror misalignment angle and the input cone angle. The imaging performance for static specimens (Figure 6c and 6d) and fast-flowing cells in microfluidic channels (Figure 6e and 6f) is generally similar to that of the SE scheme. The performance of fluorescence time-stretch microscopy based on the SE-free scheme with a line-scan rate of 8 MHz was tested using fluorescent microbeads in a microfluidic flow. The fluorescence microscopic imaging achieved at a flow speed of 2 m s^−1^ is essentially free of motion blur (Figure 6g).

These demonstrations prove the viability of both the SE and SE-free schemes for time-stretch imaging in the visible spectrum. In comparison with the SE scheme, the significance of the SE-free scheme is that narrow-band and long-pulsed lasers (for example, sub-ns) can now also be applied in this ultrafast imaging concept, in addition to the commonly adopted ultrashort broadband sources (fs–ps). This not only can make the system configuration more cost-effective but also enhances the capability of existing time-stretch imaging modalities, for example, enabling multi-color imaging in both the bright-field and fluorescence modes.

## Conclusion

We have demonstrated a new approach, FACED, that enables unprecedentedly high-quality ultrafast optical time-stretch imaging (at an all-optical laser-beam scanning rate as high as tens of MHz) in the visible spectrum—a working regime that is prevalent in conventional biological microscopy, yet has long been inaccessible in time-stretch imaging because of the scarcity of low-loss dispersive fibers for this wavelength range. In summary, the overall strength of FACED is its large-scale reconfigurability of pulse stretching (>10 ns, or equivalently, a GVD as high as >1 ns nm^−1^), which can be achieved without substantial optical loss (<6 dB). Its low loss is attributed to its free-space operating principle, which is subject to only minimal mirror reflectivity loss and is generally valid across a wide range of wavelengths (from 400 nm to as long as ~2 μm). Meanwhile, its remarkable pulse-stretching capability is made possible by the substantial light-path delay, as long as a few meters (that is, >10 ns), that is induced by the minute and adjustable mirror misalignment angle (<1 mrad) entirely in free space within a practical footprint of 100–200 × 10–20 mm (*S* × *D*) (Figure 2, Supplementary Figs. S2 and S4). Moreover, another unique attribute of FACED, which is absent in all the existing fiber-based pulse stretchers for time-stretch imaging, is the array of time-delayed virtual sources that are automatically aligned in space by the misaligned mirror geometry; in other words, FACED also intrinsically offers an ultrafast all-optical laser-beam scanning mechanism. More intriguingly, FACED also allows pulse stretching without chromatic dispersion, which has long been indispensable in conventional time-stretch imaging. This offers additional flexibility in implementing time-stretch imaging, enabling both the SE and SE-free schemes. We note that the line-scan operation of FACED-based time-stretch imaging is particularly useful for ultrafast flow imaging applications. High-speed dynamic imaging in 2D with a frame rate of up to kHz is also possible with an additional high-speed beam scanner in the orthogonal directions, such as high-speed resonant mirrors, AODs or EODs^20, 23^. Taken together, the aforementioned characteristics of FACED could drive a new paradigm of ultrafast optical time-stretch imaging in the visible spectrum. For example, we have demonstrated here that visible-light time-stretch imaging using FACED enables MHz color and fluorescence imaging—of profound interest for biological imaging, yet impossible with the current time-stretch technologies.

## References

[bib1] Gao L, Liang JY, Li CY, Wang LV. Single-shot compressed ultrafast photography at one hundred billion frames per second. Nature 2014; 516: 74–77. 25471883 10.1038/nature14005 PMC4270006

[bib2] Nakagawa K, Iwasaki A, Oishi Y, Horisaki R, Tsukamoto A et al. Sequentially timed all-optical mapping photography (STAMP). Nat Photon 2014; 8: 695–700.

[bib3] Velten A, Wu D, Adrian J, Belen M, Christopher B et al. Femto-photography: capturing and visualizing the propagation of light. ACM Trans Graphic 2013; 32: 44.

[bib4] Seo M, Boubanga-Tombet S, Yoo J, Ku Z, Gin AV et al. Ultrafast optical wide field microscopy. Opt Express 2013; 21: 8763–8772. 23571965 10.1364/OE.21.008763

[bib5] Goda K, Tsia KK, Jalali B. Serial time-encoded amplified imaging for real-time observation of fast dynamic phenomena. Nature 2009; 458: 1145–1149. 19407796 10.1038/nature07980

[bib6] Diebold ED, Buckley BW, Gossett DR, Jalali B. Digitally synthesized beat frequency multiplexing for sub-millisecond fluorescence microscopy. Nat Photon 2013; 7: 806–810.

[bib7] Schneider J, Zahn J, Maglione M, Sigrist SJ, Chojnacki J et al. Ultrafast, temporally stochastic STED nanoscopy of millisecond dynamics. Nat Methods 2015; 12: 827–830. 26214129 10.1038/nmeth.3481

[bib8] Coppinger F, Bhushan AS, Jalali B. Photonic time stretch and its application to analog-to-digital conversion. IEEE Trans Microw Theory Tech 1999; 47: 1309–1314.

[bib9] Goda K, Ayazi A, Gossett DR, Sadasivam J, Lonappan CK et al. High-throughput single-microparticle imaging flow analyzer. Proc Natl Acad Sci USA 2012; 109: 11630–11635. 22753513 10.1073/pnas.1204718109 PMC3406874

[bib10] Mahjoubfar A, Chen C, Niazi KR, Rabizadeh S, Jalali B. Label-free high-throughput cell screening in flow. Biomed Opt Express 2013; 4: 1618–1625. 24049682 10.1364/BOE.4.001618 PMC3771832

[bib11] Wong TTW, Lau AKS, Ho KKY, Tang MYH, Robles JDF et al. Asymmetric-detection time-stretch optical microscopy (ATOM) for ultrafast high-contrast cellular imaging in flow. Sci Rep 2014; 4: 3656. 24413677 10.1038/srep03656 PMC3888978

[bib12] Lau AKS, Wong TTW, Ho KKY, Tang MTH, Chan ACS et al. Interferometric time-stretch microscopy for ultrafast quantitative cellular and tissue imaging at 1μm. J Biomed Opt 2014; 19: 076001. 10.1117/1.JBO.19.7.076001 24983913

[bib13] Ugawa M, Lei C, Nozawa T, Ideguchi T, Di Carlo D et al. High-throughput optofluidic particle profiling with morphological and chemical specificity. Opt Lett 2015; 40: 4803–4806. 26469624 10.1364/OL.40.004803

[bib14] Fritzsch FSO, Dusny C, Frick O, Schmid A. Single-cell analysis in biotechnology, systems biology, and biocatalysis. Annu Rev Chem Biomol Eng 2012; 3: 129–155. 22468600 10.1146/annurev-chembioeng-062011-081056

[bib15] Hoppe PS, Coutu DL, Schroeder T. Single-cell technologies sharpen up mammalian stem cell research. Nat Cell Biol 2014; 16: 919–927. 25271480 10.1038/ncb3042

[bib16] Lei C, Guo BS, Cheng ZZ, Goda K. Optical time-stretch imaging: principles and applications. Appl Phys Rev 2016; 3: 011102.

[bib17] Lau AKS, Wong TTW, Shum HC, Wong KKY, Tsia KK. Ultrafast microfluidic cellular imaging by optical time-stretch. Imaging Flow Cytometry: Methods and Protocols. New York: Springer; 2016. pp 23–45. 10.1007/978-1-4939-3302-0_3 27460236

[bib18] Lau AKS, Tang AHL, Xu JJ, Wei XM, Wong KKY et al. Optical time stretch for high-speed and high-throughput imaging—from single-cell to tissue-wide scales. IEEE J Select Topics Quantum Electron 2016; 22: 6803115.

[bib19] Goda K, Jalali B. Dispersive Fourier transformation for fast continuous single-shot measurements. Nat Photon 2013; 7: 102–112.

[bib20] Goda K, Mahjoubfar A, Wang C, Fard A, Adam J et al. Hybrid dispersion laser scanner. Sci Rep 2012; 2: 445. 22685627 10.1038/srep00445 PMC3370333

[bib21] Marshall GF, Stutz GE. Handbook of Optical and Laser Scanning, 2nd edn. Boca Raton: CRC Press; 2011.

[bib22] Choi S, Kim P, Boutilier R, Kim MY, Lee YJ et al. Development of a high speed laser scanning confocal microscope with an acquisition rate up to 200 frames per second. Opt Express 2013; 21: 23611–23618. 24104273 10.1364/OE.21.023611

[bib23] Römera GRBE, Bechtold P. Electro-optic and acousto-optic laser beam scanners. Phys Procedia 2014; 56: 29–39.

[bib24] Tsia KK, Goda K, Capewell D, Jalali B. Performance of serial time-encoded amplified microscope. Opt Express 2010; 18: 10016–10028. 20588855 10.1364/OE.18.010016

[bib25] Diebold ED, Hon NK, Tan ZW, Chou J, Sienicki T et al. Giant tunable optical dispersion using chromo-modal excitation of a multimode waveguide. Opt Express 2011; 19: 23809–23817. 22109406 10.1364/OE.19.023809

[bib26] Qiu Y, Xu JJ, Wong KKY, Tsia KK. Exploiting few mode-fibers for optical time-stretch confocal microscopy in the short near-infrared window. Opt Express 2012; 20: 24115–24123. 23187174 10.1364/OE.20.024115

[bib27] Ahn TJ, Park Y, Azaña J. Ultrarapid optical frequency-domain reflectometry based upon dispersion-induced time stretching: principle and applications. IEEE J Select Topics Quantum Electron 2012; 18: 148–165.

[bib28] Chen HW, Lei C, Xing FJ, Weng ZL, Chen MH et al. Multiwavelength time-stretch imaging system. Opt Lett 2014; 39: 2202–2205. 24686711 10.1364/OL.39.002202

[bib29] Yazaki A, Kim C, Chan J, Mahjoubfar A, Goda K et al. Ultrafast dark-field surface inspection with hybrid-dispersion laser scanning. Appl Phys Lett 2014; 104: 251106.

[bib30] Wei XM, Lau AKS, Xu YQ, Tsia KK, Wong KKY. 28 MHz swept source at 1.0 μm for ultrafast quantitative phase imaging. Biomed Opt Express 2015; 6: 3855–3864. 26504636 10.1364/BOE.6.003855 PMC4605045

[bib31] Kärtner FX, Matuschek N, Schibli T, Keller U, Haus HA et al. Design and fabrication of double-chirped mirrors. Opt Lett 1997; 22: 831–833. 18185677 10.1364/ol.22.000831

[bib32] Shirasaki M. Large angular dispersion by a virtually imaged phased array and its application to a wavelength demultiplexer. Opt Lett 1996; 21: 366–368. 19865407 10.1364/ol.21.000366

[bib33] Golubovic B, Austin RR, Steiner-Shepard MK, Reed MK, Diddams SA et al. Double Gires–Tournois interferometer negative-dispersion mirrors for use in tunable mode-locked lasers. Opt Lett 2000; 25: 275–277. 18059853 10.1364/ol.25.000275

[bib34] Greenbaum A, Zhang YB, Feizi A, Chung PL, Luo W et al. Wide-field computational imaging of pathology slides using lens-free on-chip microscopy. Sci Transl Med 2014; 6: 267ra175. 10.1126/scitranslmed.3009850 25520396

[bib35] Amini H, Lee W, Di Carlo D. Inertial microfluidic physics. Lab Chip 2014; 14: 2739–2761. 24914632 10.1039/c4lc00128a

[bib36] Zhao YB, Tu HH, Liu Y, Bower AJ, Boppart SA. Enhancement of optical coherence microscopy in turbid media by an optical parametric amplifier. J Biophotonics 2014; 8: 512–521. 25196251 10.1002/jbio.201400073 PMC4370812

[bib37] Basiji DA, Ortyn WE, Liang LC, Venkatachalam V, Morrissey P. Cellular image analysis and imaging by flow cytometry. Clin Lab Med 2007; 27: 653–670. 17658411 10.1016/j.cll.2007.05.008 PMC2034394

[bib38] Ghaznavi F, Evans A, Madabhushi A, Feldman M. Digital imaging in pathology: whole-slide imaging and beyond. Annu Rev Pathol 2013; 8: 331–359. 23157334 10.1146/annurev-pathol-011811-120902

[bib39] Chan ACS, Wong TTW, Wong KKY, Lam EY, Tsia KK. Speed-dependent resolution analysis of ultrafast laser-scanning fluorescence microscopy. J Opt Soc Am B 2014; 3: 755–764.

