# Supplementary material for: Ultrafast laser-scanning time-stretch imaging at visible wavelengths
Source: Light Sci Appl. 2017 Jan 27;6(1):e16196–. doi: 10.1038/lsa.2016.196 (PMC6061895; doi:10.1038/lsa.2016.196)
Supplement: Supplementary Information [file lsa2016196x1.docx]

**SUPPLEMENTARY INFORMATION**

**Ultrafast Laser-Scanning Time-Stretch Imaging at Visible Wavelengths**

Jianglai Wu^1^[^†^](http://www.nature.com/lsa/journal/v5/n2/full/lsa201634a.html#note1), Yiqing Xu^1^[^†^](http://www.nature.com/lsa/journal/v5/n2/full/lsa201634a.html#note1), Jingjiang Xu^2^, Xiaoming Wei^1^, Antony C. S. Chan^1^, Anson H. L. Tang^1^, Andy K. S. Lau^1^, Bob M. F. Chung^3^, Ho Cheung Shum^3^, Edmund Y. Lam^1^, Kenneth K. Y. Wong^1^, Kevin K. Tsia^1^

*^1^Department of Electrical and Electronic Engineering, The University of Hong Kong, Pokfulam Road, Hong Kong, China.*

*^2^Department of Bioengineering, University of Washington, 3720 15th Avenue NE, Seattle, Washington 98195, USA.*

*^3^Department of Mechanical Engineering, The University of Hong Kong, Pokfulam Road, Hong Kong, China.*

[^†^](http://www.nature.com/lsa/journal/v5/n2/full/lsa201634a.html#note1)*These authors contributed equally to this work.*

**Email addresses**:

Jianglai Wu: [jianglaiwu@yahoo.com.hk](mailto:jianglaiwu@yahoo.com.hk)

Yiqing Xu: [rayxu543@gmail.com](mailto:rayxu543@gmail.com)

Jingjiang Xu: [jaysonxu@gmail.com](mailto:jaysonxu@gmail.com)

Xiaoming Wei: weixmingopt@gmail.com

Antony C. S. Chan: [cschan@eee.hku.hk](mailto:cschan@eee.hku.hk)

Anson H. L. Tang: hinlongtang@hku.hk

Andy K. S. Lau: [andyksl@hku.hk](mailto:andyksl@hku.hk)

Bob M. F. Chung: [c3m8f@hku.hk](mailto:c3m8f@hku.hk)

Ho Cheung Shum: [ashum@hku.hk](mailto:ashum@hku.hk)

Edmund Y. Lam: elam@eee.hku.hk

Kenneth K. Y. Wong: [kywong@eee.hku.hk](mailto:kywong@eee.hku.hk)

**Correspondence:** Kevin K. Tsia, Email: [tsia@hku.hk](mailto:tsia@hku.hk), Fax: (852) 2559-8738, Tel.: (852) 2857-8486

**TABLE OF CONTENT**

**I. Theory of FACED**

**1. Conjugate-mirror ray-tracing model**

**2. *Cardinal* *rays* in FACED**

**3. Pulse-stretching generated by FACED**

**4. Geometrical dependence of pulse-stretching**

**5. Virtual sources array**

**II. Beam scanning in FACED-based time-stretch imaging**

**1. *SE-free* scheme**

**2. *SE* scheme**

**III. Pulse stretching loss in FACED**

**IV. Experimental details**

**1. Basic performance tests and time-stretch imaging at 710 nm (*SE* scheme)**

**i. Experimental setups**

**ii. Basic performance tests**

**iii. Bright-field time-stretch imaging**

**iv. Fluorescence time-stretch imaging**

**2. FACED-based time-stretch imaging at 710 nm (*SE-free* scheme)**

**V. Supplementary figures**

**VI. Supplementary table**

**VII. Supplementary references**

**I. Theory of FACED**

**1. Conjugate-mirror ray-tracing model**

We present a ray-tracing model to describe the working principle of FACED. This ray-optics approach is adequate to predict the performance of the device, and thus serves as a basic and handy tool for device design and optimization. This model is valid for both *SE* and *SE-free* schemes. We consider that the input beam is first converged to the entrance *O* of the device by an angular disperser module, which primarily consist of either a diffraction grating (*SE* scheme) or a cylindrical lens (*SE-free* scheme). Figure S1 shows a schematic of a FACED device in a coordinate system in which the origin is the intersection point between planes of the two angle-misaligned mirrors (two blue lines). Each spatially-chirped zig-zag path within the FACED device can be viewed as a straight light ray passing through a series of *imaginary* plane mirrors (called *conjugate* mirrors). Each mirror makes a tilt angle *α* with respect to the neighboring mirrors, forming a fan of conjugate mirrors extrapolated from the origin. We denote *C_k_* as the line representing the *k*^th^ conjugate mirror which makes a tilt angle of *kα* with respect to the plane of *0*^th^ *mirror* *C_0_* (called *principal mirror*), which resides along the x-axis. Consider that the two mirrors in the FACED device have the same mirror length *D*; we define an outer and inner circles in the same plot, with the radii as

$R=\frac{S}{2}\left(\sin\frac{\alpha}{2}\right)^{-1}\approx\frac{S}{\alpha}$, and (S1)

$r=R-D$ (S2)

respectively. *S* is the larger separation of the two mirrors. The small-angle approximation made in Equation (S1) is valid in our case as *α* is typically small (~ 1 mrad). Adopting the vector notation, we can express the equation of line representing the *k*^th^ conjugate mirror *C_k_* as:

, (S3)

where $\vec{r}$ is the position vector (pointing from the origin), $\hat{x}$ and $\hat{y}$ are the unit directional vectors, and *k* is an integer. Similarly, we also can define the equation of line representing the *projected light ray* $l_{\theta}$ propagating through the series of conjugate mirrors as

, (S4)

where $\theta$ is the incident angle of the light ray with respect to *C_0_*.


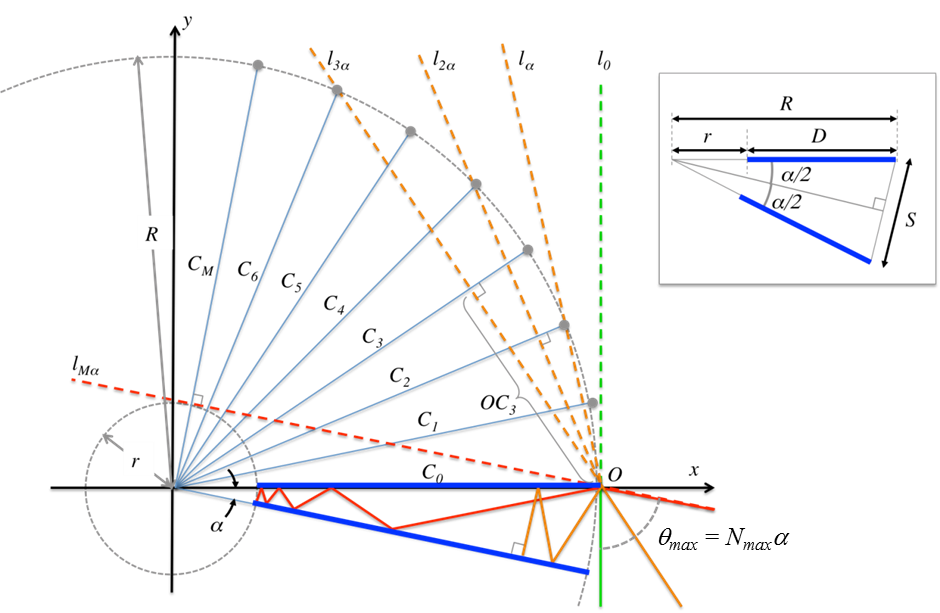


**Figure S1.** Ray-tracing diagram of a FACED device based on a conjugate-mirror model. The pair of thick blue lines represents the two angle-misaligned plane mirrors, forming the FACED device. Conjugate mirrors {*C_k_*}, defined as the images of the adjacent mirror, are represented as the thin blue lines drawn from the origin. Two light rays (solid green and red lines) are drawn to illustrate the shortest and longest spatially-chirped zig-zag paths inside the device, respectively. The green light ray makes a normal incidence on the mirror *C_0_*. The red zig-zig light ray can be “unfolded” and viewed as a straight ray intersecting the conjugate mirrors and being tangential to the inner circle (red dashed line). The point at which it touches the inner circle represents the furthest point that the light is allowed to propagate inside device and is back-reflected along the same path. The angle between the unfolded red and green lines represents the acceptance angle *θ_max_ = N_max_α *of the device, i.e. defining the device’s numerical aperture. *N_max_* is the maximum number of cardinal rays supported by the device. Three additional unfolded light paths *l_kα_* (orange dashed lines), are also included in the plot. Note that these rays *l_kα_*, because they belong to the cardinal rays, are always perpendicular to the conjugate mirrors *C_k_*. *OC_3_* is the length measured from *O* to the normal-reflection point at *C_3_*. For *k* = 1, 2, only the unfolded rays of *l_α_* and *l_2α_* are shown in the plot for clarity. The right inset shows the geometrical parameters defined for the FACED device.

**2. Cardinal rays in FACED**

One key feature of the FACED device is the ability to allow light rays to be back-reflected to the input of the device, thanks to the misaligned geometry of the mirrors giving rise to the spatially-chirped zig-zag paths. More specifically, there is a set of light paths goes back along the same pathways as the input rays. Such complete light-path reversibility has to satisfy a condition in which the projected light ray has to be orthogonal to any conjugate mirrors *C_k_*, i.e. $l_{\theta}\perp C_{k}$ (Figure S1). Based on Equations (S3) and (S4), we could deduce from this orthogonal condition:

$\begin{matrix} (\hat{x}\cos\theta+\hat{y}\sin\theta)\cdot(\hat{x}\sin k\alpha-\hat{y}\cos k\alpha)=0 \\ \Rightarrow\tan\theta=\tan k\alpha\\ \Rightarrow\theta=k\alpha. \end{matrix}$ (S5)

Hence, this condition is satisfied for a set of light rays making the incident angle at *kα* with respect to *C_0_*. We refer them as *cardinal* *rays*. The angular separation between the neighboring cardinal rays equals to *α*. The total optical path length of each cardinal ray travelling within the device is twice the distance from the entrance *O* to the corresponding conjugate mirror *C_k_* normal to the light ray *l_kα_*, named as *OC_k_* (Figure S1). We note that some light rays do not meet this orthogonal condition. We will discuss it in Section I5. Regardless the orthogonal condition, in order to make sure that the light rays can be reflected back by the FACED device, they must not enter the inner circle (i.e. the rays escape from the far end of the device). It sets a condition in which the projected light ray angle should be bounded by a maximum angle *θ*_max_, i.e. bounded by the green and red lines as shown in Figure S1. This refers to$R\cos\theta_{\max}\geq r$. We can thus define a numerical aperture (NA) of the device as

$\text{NA}=\sin\frac{\theta_{\max}}{2}=\sqrt{\frac{1-r/R}{2}}$. (S6)

It describes the maximum acceptance input cone angle within which the light rays can be back-reflected. Hence, we can estimate the maximum number of cardinal rays *N_max_* supported by the FACED device:

$N_{\max}=\frac{\theta_{\max}}{\alpha}$. (S7)

And for a given input light cone angle smaller than *θ*_max_, i.e. Δ*θ* < *θ*_max_, the corresponding number of cardinal rays is simply written as $M=\Delta\theta/\alpha$ (i.e. Equation (1) in the main text). Note that the significance of *M* or *N_max_* is that they effectively govern the number of resolvable scanned spots and thus the spatial resolution. From Equations (S1), (S2), and (S7), we note that *N_max_* can be actively adjusted by varying the geometry of the device (See the relationship between *N_max_* and the device parameters: *S*, *D*, and *α* as shown in Figure S2). In general, *N_max_* increases with smaller mirror separation for a longer mirror length, particularly in the small misaligned angle range (i.e. see top left region of the maps). On the contrary, *N_max_* decreases with wider mirror separation for a shorter mirror length, particularly in the larger misaligned angle range (i.e. bottom right region of the maps).

**
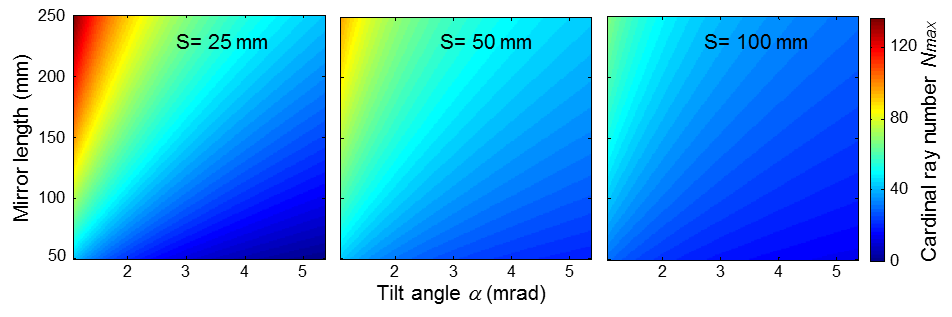
**

**Figure S2.** The dependence of the number of cardinal rays *N_max_* on the geometries of the device. The maps show that how the cardinal ray number *N_max_* varies with the mirror misaligned angle *α* and the mirror length *D*, with different mirror separations *S* = 25 mm, 50 mm, and 100 mm.

**3. Pulse stretching generated by FACED**

The temporal delay between any two cardinal rays can be directly derived from the difference of their optical paths (see Figure S1):

$\tau=\frac{2}{c}\left|\text{OC}_{k}-\text{OC}_{k'}\right|=\frac{2R}{c}\left|\sin k\alpha-\sin k'\alpha\right|\approx\frac{2S}{c}$, (S8)

where *c* is the speed of light in free space. Specifically in the *SE* scheme, the group delay dispersion (GDD) induced by the FACED device can be, by definition, written as (in a unit of s^2^) GDD = *∂^2^(ωτ) / ∂ω^2^*, where *ω* is the angular frequency of the light. Alternatively, we can quantify the dispersion in terms of wavelength, i.e. dispersion parameter (typically in ns/nm). It is simply defined as *D_λ_ = ∂τ / ∂λ*. In most cases, we are interested in the maximum temporal delay *τ_max_* supported by the device. It can be obtained by setting *k* = *N_max_* and *k'* = *0* in Equation (S8) and yield

$\tau_{\max}=\frac{2R}{c}\sin\theta_{\max}\approx\frac{2S}{c}N_{\max}$. (S9)

The approximation is taken based on Equations (S1) and (S7). When the input light cone angle is Δ*θ* < *θ*_max_, the total temporal delay can be expressed as $\Delta T_{total}\approx\frac{2S}{c}M$(i.e. Equation (2) in the main text). Again for the *SE* scheme, the total dispersion for a given total source bandwidth Δ*λ* (in wavelength), can then be evaluated as:

$D_{\lambda,Total}=\frac{\tau_{\max}}{\Delta\lambda}=\frac{2R}{c\Delta\lambda}\sin\theta_{\max}$. (S10)

**
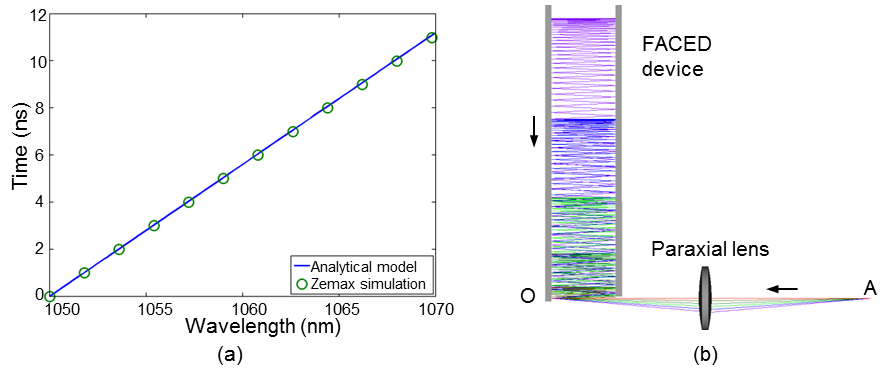
**

**Figure S3.** (a) Wavelength-to-time mapping of pulse stretching by a FACED device predicted by analytical ray-tracing model and ZEMAX simulation. (b) Ray-tracing diagram simulated in ZEMAX using the same parameters considered in the analytical model. Rays with different propagation angles from point *A* represent different wavelengths (diffracted from the diffracted grating). They are coupled into the device at *O* through a paraxial lens. The path length of each light ray is calculated as the round-trip length starting from *O*.

In particular for the case of *SE* scheme, we plot the wavelength-to-time mapping introduced by the FACED device based on our analytical ray-tracing model (Figure S3a). Here, we consider a 10-nm wide (full-width at half-maximum) transform-limited Gaussian pulse centered at 1060 nm, which has a beam diameter of 2.5 mm and illuminates on a 1200 lines/mm diffraction grating with an incident angle of 60º. Using the mirror separation *S* = 15 mm, the mirror length *D* = 200 mm, and the misaligned mirror angle *α* = 0.054^o^, we could achieve a total dispersion as large as *D_λ,total_* = 0.4 ns/nm. We can clearly observe that the calculated wavelength-to-time mapping is linear and is also consistent with the numerical ray-tracing simulation using ZEMAX, which adopts the same set of parameters (Figure S3b).

**4. Geometrical dependence of pulse-stretching**

Based on the above formulation, we further investigate the reconfigurability of pulse-stretching (or dispersion in the *SE* scheme) by the geometrical parameters of the device. For the sake of argument, we present the study in terms of dispersion parameter *D_λ,total_* by considering a source bandwidth of 10 nm for the *SE* scheme. For the *SE-free scheme*, the total pulse stretching can simply be converted to 10*D_λ,total_*. (in ns). Figure S4 shows the general trend that dispersion increases with mirror separation *S* and is independent of the mirror length. However, it tends to more easily result in a loss of spectrum in the case of wider mirror separation due to the insufficient mirror length, i.e. the “unfolded” light ray enters the inner circle region before it is normal-reflected. It manifests in the bottom left corner regions of the middle and right maps. Again, in order to mitigate the loss of spectrum, one should reconfigure the diffraction grating as well as the telescopic relay-lens module in such a way that the NA can accommodate the entire bandwidth. These graphs provide a useful guidance for the design and optimization of pulse stretching in FACED.


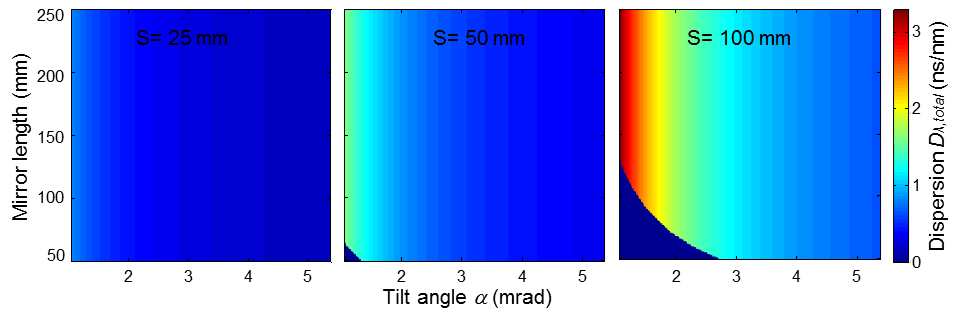


**Figure S4.** Maps of *D_λ,total_* as a function of misaligned mirror angle *α* and mirror length *D* for different mirror separations (left to right): *S* = 25 mm, 50 mm, and 100 mm. For the *SE-free* scheme, the total pulse stretching can simply be converted to 10*D_λ,total_*. (in ns). The dark blue region in the *D_λ,total_* maps represents the scenario in which part of the spectrum is lost. It is due to that some light rays leak out from the far end of the mirror, i.e. the “unfolded” light ray enters the inner circle region before it is normal-reflected.

**5. Virtual sources array**

Regarding the light rays that do not belong to the set of cardinal rays, they still follow the spatially-chirped zig-zag paths and can also be back-reflected, but without following the original path. We here show that these rays, along with the cardinal rays, can be viewed as if they emerge from a group of virtual point sources. In Figure S5, the ray in orange shows the *k*^th^ cardinal ray. After *k* reflections, it hits the mirror at normal incidence and reverses its path. With another *k* reflections, it leaves the entrance *O*, following the original path. This ray can be alternatively viewed as if it originates from *O_k_*, which is the conjugate point of the entrance *O* with respect to *C_k_*. For the rays deviates from the cardinal rays but subject to the same number of reflections before leaving the device (within $\angle AO_{k}A^{'}$), i.e. same number of intersections with the conjugate mirrors, they can be traced back to the same virtual source *O_k_*, and hence can also be viewed as if they are from *O_k_*.

As illustrated by the green and red rays in Figure S5, the output cone of *O_k_* is given by $\angle AO_{k}A^{'}=\angle AO_{k}O$ +$\angle A^{'}O_{k}O$. Using the law of sines in the triangle *AOO_k_*, we have

$\frac{AO}{\sin(\angle AO_{k}O)}=\frac{AO_{k}}{\sin(\angle AOO_{k})}$ (S11)

where $AO=S=2Rsin(\alpha/2)$; $AO_{k}=2Rsin(k\alpha+\alpha/2)$; $\angle AOO_{k}=180-k\alpha-\alpha/2$. Then$\angle AO_{k}O$ is $\alpha/2$. Similarly in the triangle *A'OO_k_*, we have

$\frac{A^{'}O}{\sin(\angle A^{'}O_{k}O)}=\frac{OO_{k}}{\sin(\angle OA^{'}O_{k})}$ (S12)

where $A^{'}O=S=2Rsin(\alpha/2)$; $OO_{k}=2Rsin(k\alpha)$; $\angle OA^{'}O_{k}=180-k\alpha$. Then $\angle A^{'}O_{k}O$ is $\alpha/2$. It is thus clear that each virtual source *O_k_* carries a light cone angle of *α *.


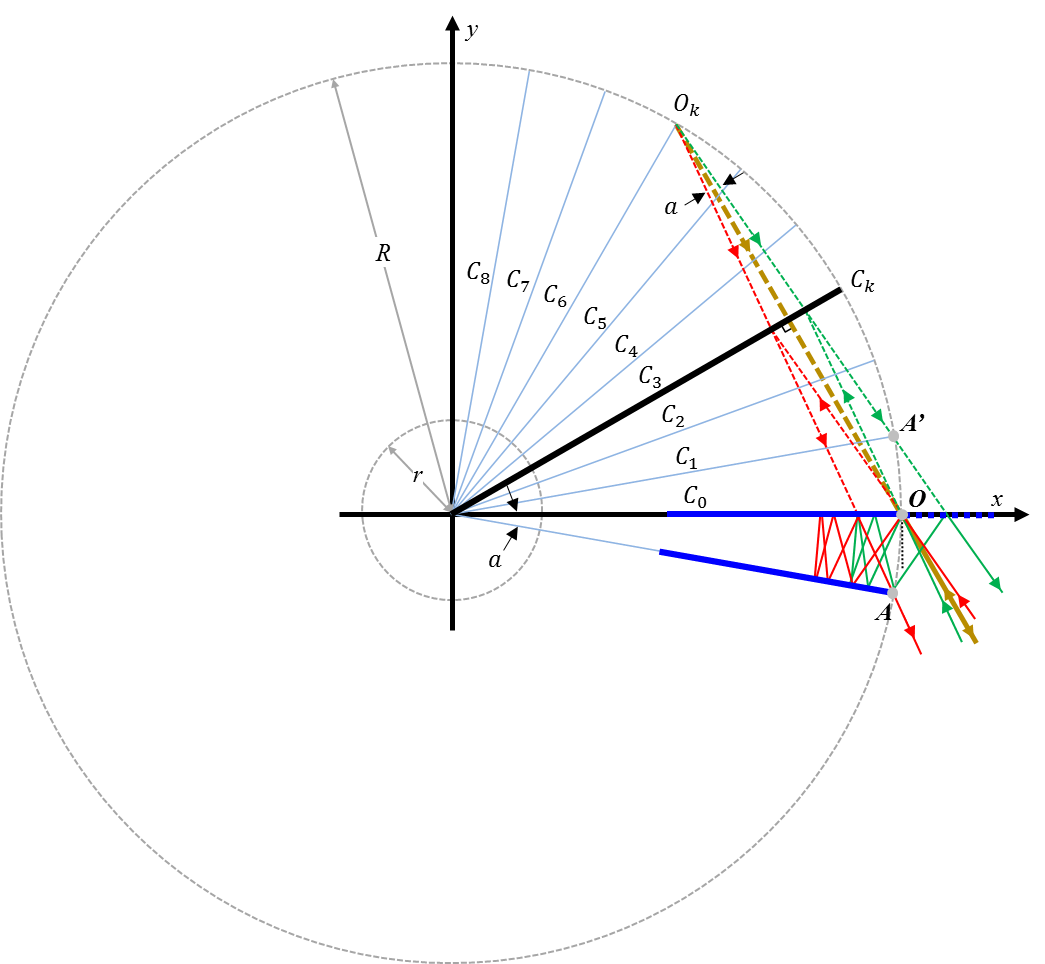


**Figure S5.** Illustration of a virtual source in FACED. The orange ray shows the *k^th^* cardinal ray. *O_k_* is the conjugate point of *O* with respect to *C_k_*, i.e. the virtual source. The red and green rays are the upper and lower bound of the light rays such that they are still subjected to the same number of mirror reflections (= *k* in this case) as that of the *k*^th^ cardinal ray. We here assume the mirror length is extended beyond entrance *O* (see blue dashed line) in order to ensure all the light rays is reflected back from the device.

**II. Beam scanning in FACED-based time-stretch imaging**

We here investigate the considerations to manipulate the virtual sources for beam-scanning in FACED-based time-stretch imaging in both the *SE-free* and *SE* schemes.

**1. *SE-free* scheme**


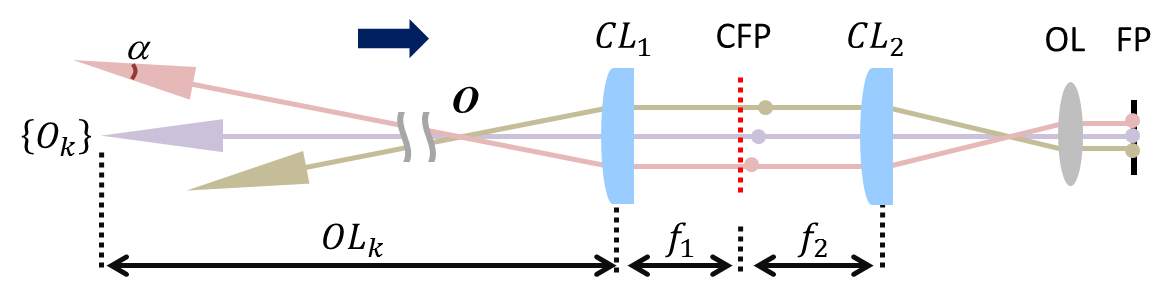


**Figure S6.** *SE-free* scheme based time-stretch imaging using FACED device. Cylindrical lens (CL_1_, CL_2_), common focal plane (CFP), objective lens (OL), focal plane (FP), object length of virtual source *O_k_* to *CL_1_* (*OL_k_*).

In the *SE-free* scheme, virtual source *O_k_* can be viewed as a point source carrying a cone angle of *α* (Figure S6). It is first imaged by the cylindrical lens and is further relayed to the focal plane of the microscope. Under the consideration of the device $NA\ll1$ (typical input cone < 5º), the distance between *O_k_* to the cylindrical lens (*CL_1_*) can be approximated to

. (S13)

The image length $OL_{k}^{'}$can then be expressed as

$OL_{k}^{'}=f_{1}(1+\frac{f_{1}}{2kS})$, (S14)

which suggests that the virtual sources are imaged at different image planes. Nevertheless, by manipulating the *f_1_*, *k*, and *S*, all the virtual sources can be imaged in the proximity of the CFP and are within the focal depth of the infinity-corrected microscope for time stretch imaging, i.e. all the virtual sources are imaged within the depth-of-field of the microscope. The angular magnification factor for *O_k_* by the coupling cylindrical lens is:

$AM_{k}=OL_{k}/OL_{k}^{'}=2kS/f_{1}$, (S15)

Then the beam size of the corresponding virtual sources *O_k_* at the back aperture of the objective lens is:

$BS_{k}\approx AM_{k}\times\alpha\times f_{2}=2kS\alpha f_{2}/f_{1}$ (S16)

The beam size is proportional to *k*. We can make the beam size of the first virtual source (smallest *k*) just fill the back aperture of the objective lens whereas all the other virtual sources overfill the aperture. Such configuration takes full use of the resolving power of the objective lens at the cost of power and illumination uniformity.


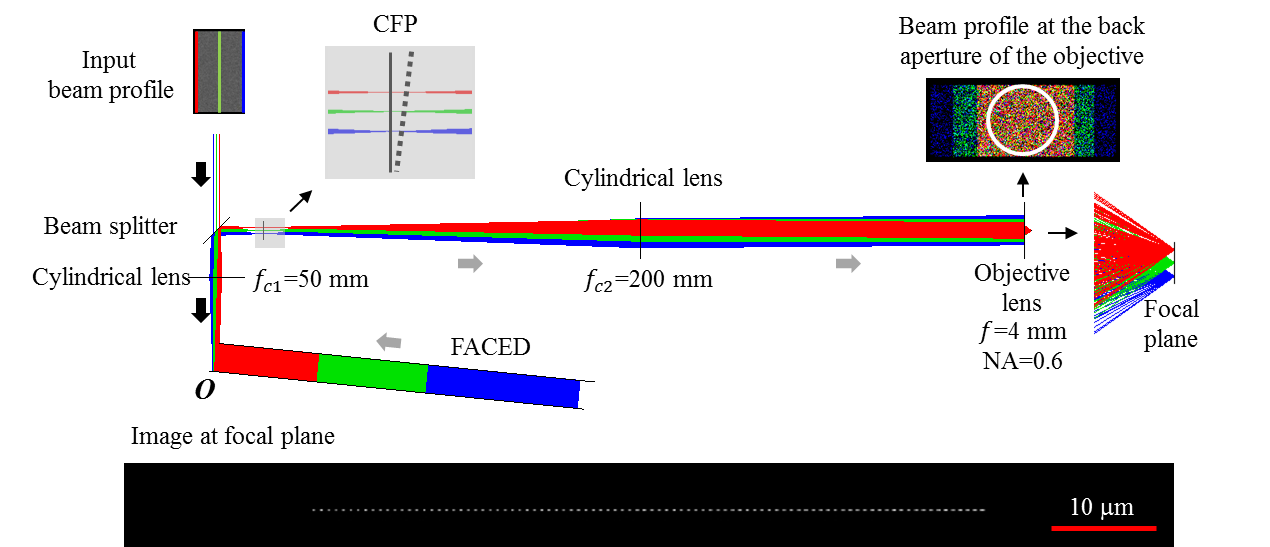


**Figure S7.** Ray-tracing simulation of the *SE*-free scheme using Zemax. Key parameters of the FACED device: *D* = 200 mm, *S*= 15 mm, *α =* 0.04*°.* A collimated and rectangular shaped beam (6 mm×3.2 mm) is coupled into the device to generate the virtual sources array which are imaged to the focal plane of the infinity-corrected microscope (50×, NA = 0.6). The rays in red, green, and blue show the tracing of the virtual source *O_100_* to *O_145_*, and *O_190_*.

As an example, we simulate a practical FACED device for generating an all-optical scanner (Figure S7). The tilt angle *α* = 0.04*°,* hence the NA is 0.076 (*θ_max_* = 7.8°) and can accommodate *N_max_*~ 195 cardinal rays (virtual sources). The input cone angle is Δ*θ* = 3.6° and *M* = 90 virtual sources are obtained. The virtual sources start from *O_100_* end to *O_190_* and are imaged near the CFP with a position difference along the optical axis smaller than 1 mm (by Equation (S14)). The 1-mm image depth is coupled to the depth-of-field of the microscope (50×/ 0.6). Image of resulted all-optical scanner is shown at the bottom of Figure S7. In general, the 90 virtual sources are uniformly distributed. The beam profiles of virtual source *O_100_*, *O_145_*, and *O_190_* at the back aperture of the objective lens show that all the virtual sources can fill the aperture (white circle, 6 mm in diameter). This is essential to take advantage of the resolving power of the objective lens and to ensure that the resolving power is uniform across the field-of-view.

**2. *SE* scheme**

In principle, Equations (S13)-(S16) are also valid in the *SE* scheme. The scanning beam pattern generated in this scheme has however some subtle differences when compared with the *SE-free* scheme. It is due to the fact that the input light to the FACED device originates from the angular dispersion of a collimated beam diffracted by the diffraction grating. In effect, each angular component in the virtual source can be regarded as a collimated beam with a finite beam size (Figure S8). Consequently, the virtual sources are imaged near the CFP as an elongated spots by the lens (L_1_). The length (major axis) of the elongated spots approximately equals to the production of the *f_1_* and *α*. Decreasing *α* decreases the length of the elongated spots near the CFP; the spot can be further demagnified by the microscope and converged to the diffraction limit. We here simulate the effect of *α* on the generated all-optical scanner using *SE* scheme. The FACED device has *D* = 200 mm, *S* = 15 mm. Input beam diameter is 6 mm. After grating, the beam is dispersed and cone angle is 3.6° and 1:1 relayed to the device entrance *O*. The virtual sources are first imaged by the lens (*f_1_* = 50 mm) and further relayed to the infinity-corrected microscope (tube lens, *f_2_* = 200 mm; objective lens *f* = 4 mm, NA = 0.6).


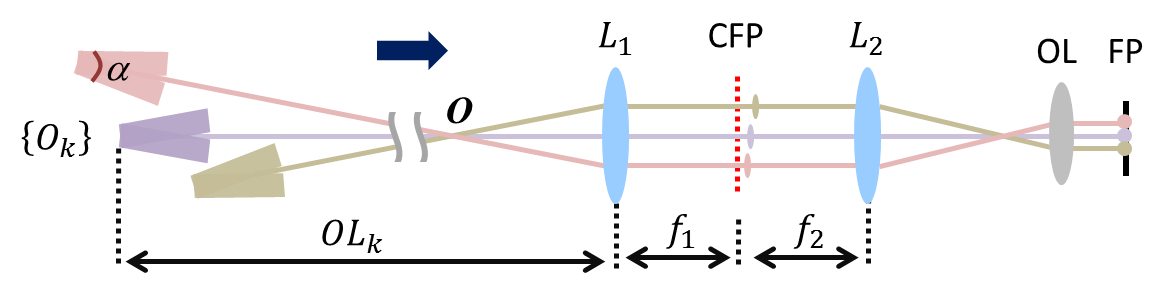


**Figure S8.** *SE* scheme based time-stretch imaging using FACED device. Lens (L_1_, L_2_), common focal plane (CFP), objective lens (OL), focal plane (FP), object length of virtual source *O_k_* to L_1_ (*OL_k_*).


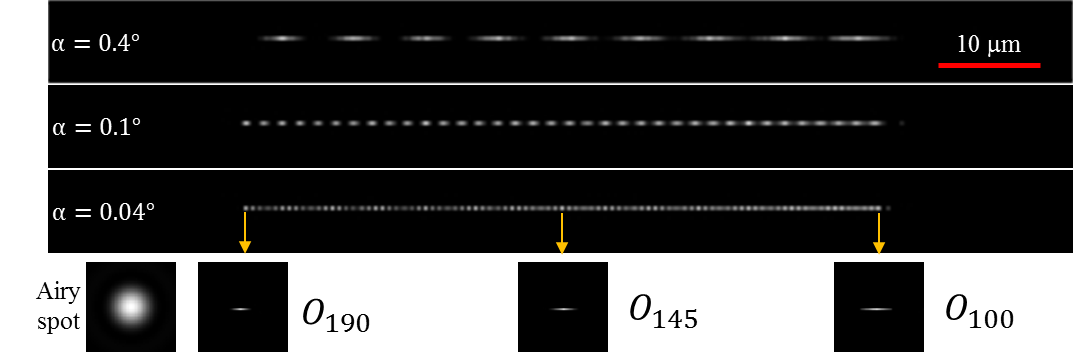


**Figure S9.** Ray-tracing simulation of beam-scanning based on the *SE* scheme using Zemax. Note that in the vertical dimension, the virtual sources keep collimated before entering the objective lens and hence perfectly focused in the ray tracing; the FACED does not change the beam profile along the vertical dimension.

Figure S9 shows the images of the scanned spots on the focal plane at 3 different misaligned mirror angles. Decreasing *α* increases the number of spots and decreases the spot size. At *α =* 0.04*°*, 90 virtual sources, start from *O_100_* end to *O_190_*, are generated. Increasing *k* decreases the spot size because the virtual sources are further away from the entrance O, and can be viewed as collimated beam. The spot sizes of these virtual sources, however, are all smaller than the Airy spot generated by the same objective lens, which suggests diffraction limited resolution can be achieved.

**III. Pulse stretching loss in FACED**

As the temporal delay is introduced entirely in free space, the key intrinsic loss of FACED is attributed to the less-than-unity mirror reflectivity Γ. Assuming both mirrors have the same reflectivity Γ and considering the total incident power is uniformly distributed across the spectral shower beam, and thus across the *M* cardinal rays, we can estimate the intrinsic loss, loss solely due to the mirror reflectivity, of the FACED device based on the ray-tracing diagram shown in Figure S1. It can be written as:

$Loss=\frac{1}{M}(\Gamma+\Gamma^{3}+\Gamma^{5}+...+\Gamma^{2M-1})=\frac{1}{M}\frac{\Gamma(1-\Gamma^{2M})}{1-\Gamma^{2}}$. (S17)

Consider the low-order approximation, *Loss ≈* Г*^M^* when Γ approaches to 1 for large *M*. This is the case in our experiments reported in this paper, e.g. Γ > 0.995 and *M* is typically ~ 100 or above. Therefore, in the *SE* scheme, we could evaluate an important figure-of-merit (FOM, in a unit of ns/nm/dB) of FACED for pulse stretching (using Equations (S1)-(S10)), i.e. dispersion-to-loss ratio:

$\text{FOM}_{\text{SE}}=\frac{D_{\lambda,Total}}{Loss}\approx\frac{2R\sin\Delta\theta}{c\Delta\lambda M\left|10\log_{10}(\Gamma)\right|}\approx\frac{2S}{c\Delta\lambda\left|10\log_{10}(\Gamma)\right|}$. (S18)

Note that in *SE-free* scheme, the FOM (ns/dB) is simply

$\text{FOM}_{\text{SE-free}}=\frac{\Delta T_{total}}{Loss}\approx\frac{2S}{c\left|10\log_{10}(\Gamma)\right|}$ (S19)

Clearly, the FOMs in both schemes can be optimized by manipulating the mirror separation, and mirror reflectivity. For the sake of argument, we show the analysis of FOM in the context of the *SE* scheme, as illustrated in Figure S10. This estimation is able to predict the trend of FOM at the three wavelengths with different bandwidths (see the three highlighted points in Figure S10b). Figures S10d – S10f show the experimentally measured loss of FACED at different dispersion, at 710 nm, 1060 nm, and 1550 nm, respectively. Note that the FOM decreases with the bandwidth of the pulsed laser.


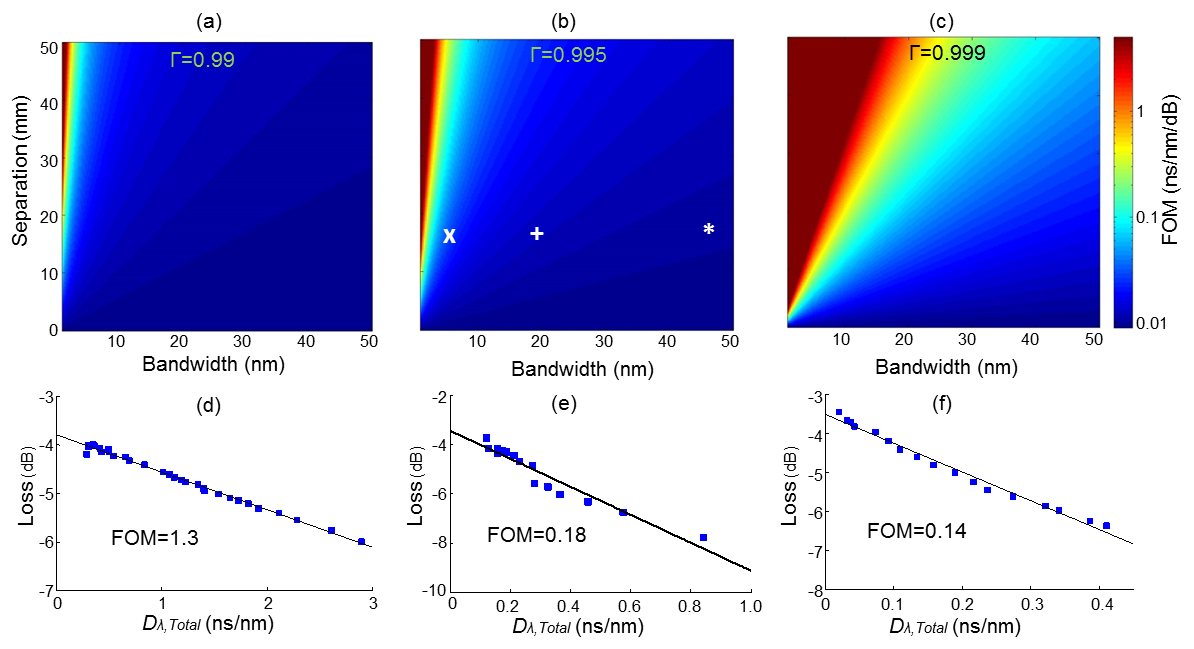


**Figure S10.** (a) - (c) Maps of FACED’s figure-of-merit (FOM) in the *SE* scheme, i.e. dispersion-to-loss ratio, as a function of the mirror separation *S*, and the bandwidth of the light source for reflectivity (Г) of (a) 0.99, (b) 0.995 and (c) and 0.999. (d)-(f) Experimental measurements of the device’s intrinsic loss as a function of dispersion (*D_λ,Total_*) (blue square dots) at different wavelengths centered at (d) 710 nm, (e) 1060 nm, and (f) 1550 nm. The black lines in the plots are the linear fits; the FOM equals the reciprocal of the slop in the linear fit. The bandwidths of laser sources are, 5 nm for the 710 nm source (indicated as “×” in (b)), 20 nm for the 1060 nm source (indicated as “+” in (b)), and ~ 47 nm for the 1550-nm source (indicated as “*” in (b)).

**IV. Experimental details**

**1. Basic performance tests and time-stretch imaging at 710 nm (*SE* scheme)**

**i. Experimental setups**

As shown in Figure S11, a femtosecond laser beam is first spatially dispersed by the diffraction grating G1, and then is coupled into the FACED device through a 4-f lens system formed by the lenses L1 and L2. The pulses are time stretched by and back reflected from the device. Note that the collected spectral shower is essentially an ultrafast 1-D line-scan beam (because of the time-stretch process) on the common focal plane (CFP). An infinity-corrected microscope, formed by objective lens O1 and tube lens L3, further de-magnifies (40×) the spectral shower and projects it onto the specimen plane, which is the conjugate plane of CFP. The iris diaphragm located at the CFP acts as a spectral filter controlling the input spectral bandwidth to 4 – 5 nm and the field-of-view to ~ 50 μm. A video camera is used to observe the beam profile and specimen at focal plane.

**
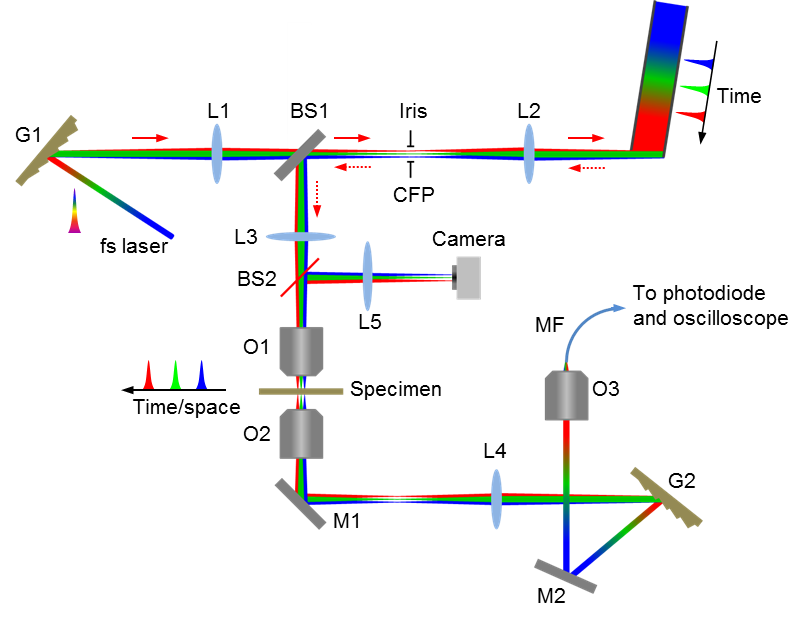
**

**Figure S11.** Experimental setup for basic performance tests of FACED (in Figure 2) and time-stretch microscopy at 710 nm (in Figures 3 – 5). The key components include: a femtosecond pulsed laser centered at 710 nm (MaiTai BB, Newport Inc.), with a pulse width of ~ 150 fs, a repetition rate of 80 MHz, an average output power of 500 mW, a bandwidth of 10 nm and a beam diameter of 4 mm; two diffraction gratings G1 and G2 (both with the groove density of 1800 lines/mm); the objective lenses O1, O2, and O3: 40× / 0.66, 20× / 0.4, and 10× / 0.25. L1, L2, L3, L4, and L5 are plan-convex lenses with the focal lengths of 75, 50, 200, 100, and 125 mm, respectively. BS1 and BS2 are pellicle beam splitters ([BP145B1](https://www.thorlabs.hk/thorproduct.cfm?partnumber=BP145B1) and BP108, Thorlabs Inc.). MF is a 1-meter multimode fiber with a core diameter of 62.5 μm. CFP is the common focal plane of L1 and L2. For fluorescence time-stretch imaging, the collection optics (after specimen) is replaced by objective lens (40× / 0.66), bandpass filter, and photomultiplier tube. The FACED device is formed by two identical high-reflectivity dielectric mirrors (height × length: 25 mm × 200 mm) separated by 15 mm; the reflectivity of the mirrors is > 99.5% near 710 nm (ios™ Optics).

**ii. Basic performance tests**

For the experiments that test the basic performance of the device (as shown in Figure 2), a multimode fiber is directly positioned on the specimen plane after the objective lens O1 in order to collect the time stretched pulses. Two optical fiber needles are located on the CFP as the spectral mask to reshape the spectrum, i.e. to generate two spectral dips in the spectra and thus the time waveforms (See Figure 2). This mask is essential for calibrating and optimizing the dispersion generated by the FACED device. The time waveforms are detected by the high-speed photodiode (electrical bandwidth 10 GHz, Picometrix) and real-time oscilloscope (Agilent DSO9404A). Note that the relatively lower bandwidth of the oscilloscope helps smooth the sub-pulse features, without digital filtering, whereas the overall spectral (temporal) features are still preserved. The optical spectrum analyzer (OSA) (Agilent 86142B) is used to measure the spectra (Figure 2e) as the reference to the time-stretched waveforms and it is only compatible with an input fiber of core size of 9 μm. Currently, the multimode fiber (core diameter of 62.5 μm) is connected to the OSA input for directly collecting the transmitted 1-D spectral shower. As a result, the multimode fiber effectively introduces additional chromomodal dispersion effect which is then translated to the wavelength dependent loss (see Figure 2e and Figure S14b) as the light is coupled to the OSA input, because of the mismatch of the fiber core size (62.5 μm to 9 μm).

The misaligned mirror angle *α *is controlled by a calibrated rotational stage equipped with a differential micrometer (Newport 481-A). By rotating the stage, we can actively tune the dispersion in large scale, as shown in Figure 2. To characterize the loss of the device, the input laser power is measured after lens L2 and before the device; the output power is measured after the beam splitter (BS1) and at the focal plane. The intrinsic loss of the device is evaluated as the measured loss excluding the ~ 3 dB loss due to the beam splitter (Figure 2g).

**ii. Bright-field time-stretch imaging**

In the experiments of visible-light time-stretch microscopy based on FACED, the light transmitted through the specimen, either tissue section fixed on the glass slides or isolated cells in microfluidic flow, is collected with the objective lens O2 and is collimated by lens L4. The spectrally-encoded beam is then recombined by the grating G2, followed by being coupled into a multimode fiber with the objective lens O3 (Figure S11). The space-encoded signals are finally detected by the high-speed photodiode and real-time oscilloscope (Agilent DSO-X 91604A or DSO9404A). The mirror separation *S* is fixed at 15 mm and the number of virtual sources is optimized to be ~ 120 across the field-of-view of 50 μm. To prevent cross talk between image lines, note that the maximum number of virtual sources is ~ 125, which is limited by the repetition rate of the pulsed laser (80 MHz).

To image static samples (e.g. tissue sections fixed on the glass slides), we use a motorized transitional stage (Newport LTA-HS) to scan (slow-axis) the sample orthogonal to the 1-D spectral shower (fast-axis). The scan step size is set to be 200 nm. At each step, an image line is recorded from the average of 8 pulses, resulting in an effective line-scan rate of 10 MHz. To visualize the larger field-of-view, we digitally stitch three to four 2D images along the fast-axis (e.g. Figures 3d, 5b, and 5d). By simply tilting the spectral shower illumination onto the specimen, one can switch between the bright-field time-stretch imaging mode (e.g. Figure 3d) and the ATOM mode, i.e. exhibiting phase-gradient contrast (e.g. Figure 5b) ^1^.

To image live cells in ultrafast microfluidic flow, we flow the cells, e.g. red blood cells, leukemic monocytes (THP-1) and microphytoplankton, at ~ 2 m/s, through a microfluidic channel in which inertial lift force balance against viscous drag force such that the cells flow in a single file. The imaged cells are flowing in the direction orthogonal to the spectral shower which is line scanned at a rate up to 80 MHz, governed by the laser repetition rate. The flow motion together with the all-optical time-stretch scanner generates 2-D images.

Detailed descriptions of the design and fabrication of the microfluidic channel can be referred to the previous work^1^. In brief, the microfluidic chip consists of two parts: a focusing section followed by an imaging section. The focusing section consists of multiple pairs of connected curved channels with radii of curvature 400 µm and 1000 µm, respectively (16 turns in total). The width (150 µm) and height (30 µm) of the channel were chosen such that the channel is suitable for focusing cells with a size ranging from ~ 5 – 20 µm. In the imaging section where the spectral shower is illuminated onto, the channel width is narrowed to 60 µm to further boost the flow speed. Note that laminar flow condition is still satisfied at such ultrafast flow (Reynolds number < 100).

**iii. Fluorescence time-stretch imaging**

The illumination optics in fluorescence time-stretch microscope is identical to that in bright-field time-stretch microscope (Figure S11). The detection optics employs objective lens (40× / 0.66) to collect the transmitted fluorescence and bandpass filter (FF01-769/41-25, Semrock Inc.) to reject the transmitted and scattered excitation laser light. The fluorescence is detected with a photomultiplier tube (PMT) (H10721-20, Hamamatsu). The output signal from the PMT is further amplified with preamplifier (PA200-10, Photek Ltd.) and is digitized with the high-speed real-time oscilloscope (Agilent DSO-X 91604A). To demonstrate fluorescence time-stretch microscopic imaging, tissue papers stained with fluorescent dyes (Antibody-CF™750 conjugates, 100 μg/ml, Sigma-Aldrich) are imaged. The sample is orthogonally scanned through the 1-D spectral shower with the transitional stage as in the bright-field time-stretch microscope. The scan step size is 500 nm. We take an average of 64 pulses at each step for each image line, resulting an effective line-scan rate of 1.25 MHz.

**4. FACED-based time-stretch imaging at 710 nm (*SE-free* scheme)**


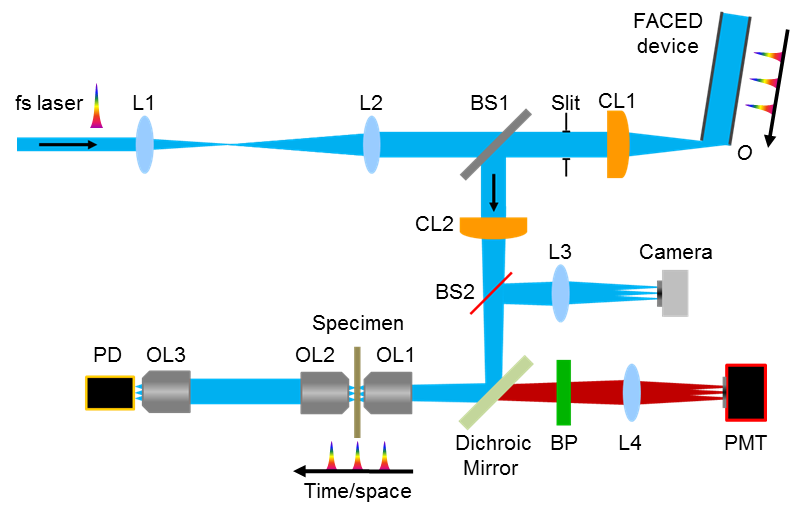


**Figure S12.** Experimental setup for FACED-based time-stretch imaging at 710 nm under the *SE-free* scheme. The laser and FACED device is the same as that in the *SE* scheme work. The objective lenses O1, O2, and O3: 40× / 0.75, 10× / 0.25, and 10× / 0.25. L1, L2, L3, L4: plan-convex lenses with the focal lengths of 25, 100, 125, 150 mm. CL1, CL2: cylindrical lenses with focal lengths of 50 mm and 250 mm. PD: photodiode. BP: bandpass filter. BS1 and BS2 are pellicle beam splitters ([BP145B1](https://www.thorlabs.hk/thorproduct.cfm?partnumber=BP145B1) and BP108, Thorlabs Inc.).

The femtosecond laser beam is first expanded by the telescope formed by L1 and L2 (5×). A slit (width ~ 3 mm) is used to select the central part of the Gaussian beam (~ 8 mm in diameter) and also to limit the field-of-view for time-stretch imaging to ~ 50 μm (Figure S12). The beam then is focused to the FACED device by a cylindrical lens (CL1). The virtual source array is imaged by the microscope, formed by cylindrical lens (CL2) and objective lens (OL1), onto the focal plane. The light transmitted through the specimen is collected by the objective lenses (OL2, OL3) and a high-speed photodiode (ET-4000A, EOT). The space-encoded temporal signals are sampled by the real-time oscilloscope (Agilent DSO-X 91604A). The mirror separation is fixed at 13.5 mm and the number of virtual sources is optimized to be ~ 70 across the field-of-view of 50 μm. The video camera is used to observe the beam profile and specimen at focal plane. The procedures to image static samples and flowing cells are the same as that in the *SE* scheme.

The objective lens (OL1), dichroic mirror (FF740-Di01, Semrock Inc.), bandpass filter (FF01-769/41-25, Semrock Inc.), and lens (L4) form an infinity-corrected fluorescence microscope for time-stretch fluorescence imaging. The fluorescence signal is detected by the PMT as in the *SE* scheme. To image the fluorescent beads in flow (Figure 6g), the mirrors’ separation is fixed at 300 mm (delay between adjacent scanned spots is 2 ns) and the number of scanned spots is optimized to be ~ 60 across the field-of-view of 50 μm. To prevent crosstalk between adjacent scanning lines, a pulse picker decreases the laser repetition rate from 80 MHz to 8 MHz (line scan rate). The procedures of flowing bead imaging are the same as that in the *SE* scheme.

**V. Supplementary figures**


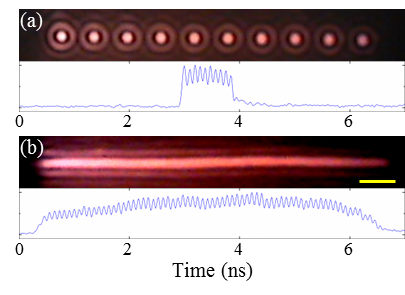


**Figure S13.**  Images of the of the virtual source arrays on the focal plane in time-stretch microscope under the *SE-free* scheme at 710 nm: (a) 10 virtual sources and (b) ~ 70 virtual sources. Scanning pattern in (b) is used for time-stretch imaging. The bottom graphs show their corresponding time waveforms. Scale bar is 5 μm.


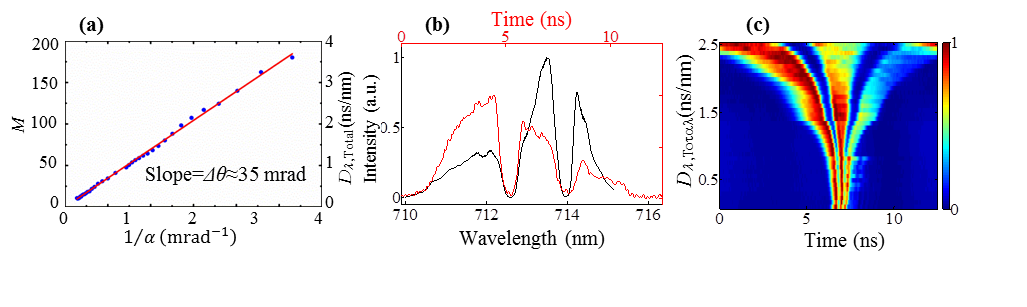


**Figure S14.** Anomalous dispersion generated by FACED under the *SE* scheme. (a) Dependence of the number of cardinal rays and total dispersion on the reciprocal of tilt angle (1/*a*). Blue dots indicate the measured data and red line shows the linear fit. The slope of the fit equals the light input fan angle *Δθ*. (b) Single-shot stretched waveform (red) and the corresponding spectra (black) measured by the conventional spectrometer with *D_λ,Total_* = +2 ns/nm. (c) Evolution of temporal profile of the stretched pulse within *D_λ,Total_* = +200 ps/nm and +2.5 ns/nm.

**
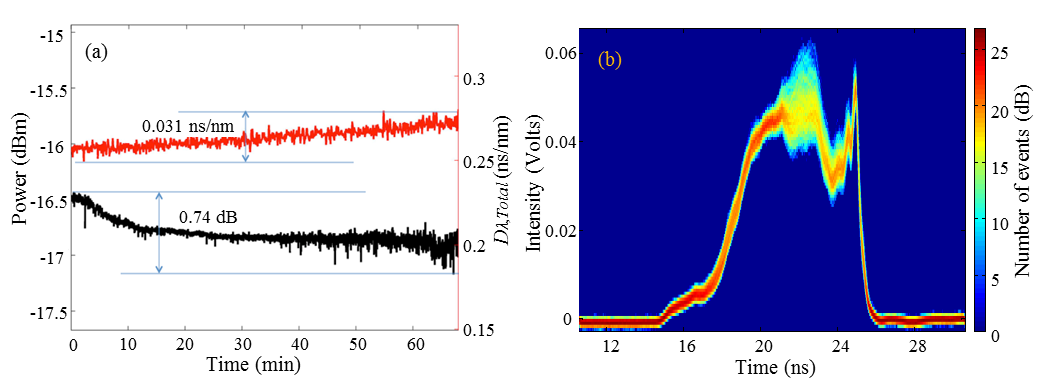
**

**Figure S15.** Long-term stability of FACED. (a) Measurement of dispersion (red) and power (black) over 1 hour. Power drift: 2.4% (std/mean); dispersion drift: 2% (std/mean). (b) 800 overlapped single-shot, real-time stretched waveforms measured over the same time period. The color scale shows the number of events (pulses). The light source is a homebuilt all-fiber broadband mode-locked laser based on stretched-pulse additive pulse mode-locking (APM) ^2,3^. Repetition rate = ~ 11.5 MHz; center wavelength = 1555 nm. The mirror pair employed here has the high-reflectivity (> 99.5%) spectra centred at 1550 nm. Mirror length (*D*) and separation (*S*) are 200 mm and 15 mm, respectively.

**
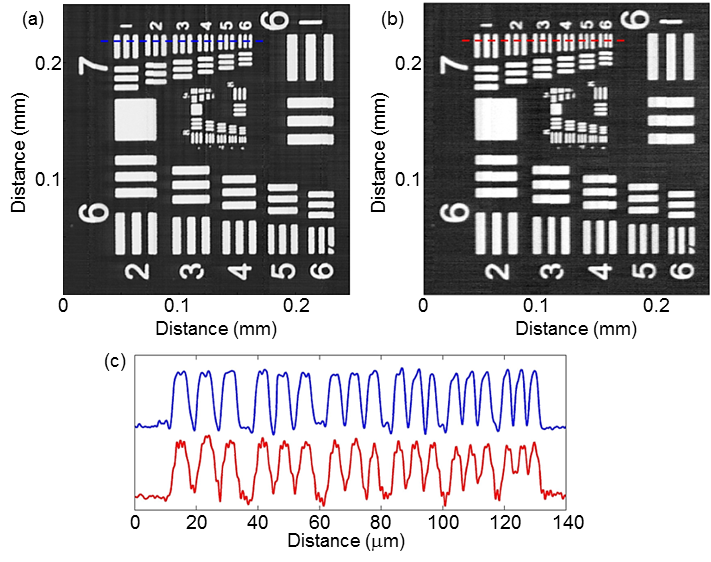
**

**Figure S16.** Optical time-stretch imaging (at 1064 nm) of resolution target (USAF-1951) using (a) a dispersive fiber (dispersion of ~ 0.45 ns/nm) and (b) a FACED device (dispersion of 0.93 ns/nm). (c) The line intensity profiles (highlighted in (a) and (b)) in the case of using the dispersive fiber (blue) and the FACED device (red). The laser source is a home-built ytterbium-doped fiber mode-locked laser (repetition rate = 26 MHz; center wavelength = 1064 nm) with a 3-dB bandwidth of ~ 10 nm and a pulse width of 4 ps^1^. We use a motorized transitional stage (Newport LTA-HS) to scan (slow-axis) the sample orthogonal to the 1-D spectral shower (fast-axis). Hence, a 2-D time-stretch image (with single-shot line-scans at a rate of 11.5 MHz) can be captured. The scan step size is set to be 200 nm. The final 2-D images (b) are formed by digitally stitching 5 raw 2D images along the fast axis – resulting in a total field-of-view of 0.25 mm × 0.25 mm. In the 1060-nm setup, we route the time-stretched beam, after back-reflected from the FACED device and recombined by the diffraction grating, to the spectrally-encoded imaging system with the configuration following our prior work^1^.

**VI. Supplementary table**

| Table S1. Major experimental parameters adopted in different FACED experiments | | | | | | |
| --- | --- | --- | --- | --- | --- | --- |
|  | **Laser source** | | **FACED device** | | **Detector** | **Oscilloscope** |
|  | **Wavelength** | **Bandwidth** | **Mirror length** | **Mirror separation** |  |  |
| Figures 2d – 2g ,  Figure 3, Figure 5, Figure S14 | 710 nm | 5 nm | 200 mm | 15 mm | 10 GHz | 4 GHz, 20 GSa/s |
| Figure 2b, Figure 4 | 710 nm | 5 nm | 200 mm | 15 mm | 10 GHz | 16GHz, 80 GSa/s |
| Figure 6, Figure S13 | 710 nm | 10 nm | 200 mm | 13.5 mm | 9 GHz | 16 GHz, 80 GSa/s |
| Figure S15 | 1555 nm | 30 nm | 200 mm | 15 mm | 10 GHz | 16 GHz, 80 GSa/s |
| Figure S16 | 1064 nm | 10 nm | 200 mm | 15 mm | 10 GHz | 16 GHz, 80 GSa/s |

**VII. Supplementary references**

1. Wong TTW, Lau AKS, Ho KKY, Tang MYH, Robles JDF *et al*. Asymmetric-detection time-stretch optical microscopy (ATOM) for ultrafast high-contrast cellular imaging in flow. *Sci Rep* 2014; **4:** 3656.
2. Ippen EP, Haus HA, Liu LY. Additive pulse mode locking. *J Opt Soc Am B* 1989; **6**:1736-1745.
3. Wei X, Xu J, Xu Y, Yu L, Xu J *et al*. Breathing laser as an inertia-free swept source for high-quality ultrafast optical bioimaging. *Opt Lett* 2014; **39**: 6593-6596.
